# BiCLUM: Bilateral contrastive learning for unpaired single-cell multi-omics integration

**DOI:** 10.1371/journal.pcbi.1013932

**Published:** 2026-02-03

**Authors:** Yin Guo, Izaskun Mallona, Mark D. Robinson, Limin Li

**Affiliations:** 1 School of Mathematics and Statistics, Xi’an Jiaotong University, Xi’an, Shannxi, China; 2 Department of Molecular Life Sciences and SIB Swiss Institute of Bioinformatics, University of Zurich, Zurich, Switzerland; University of Wisconsin Madison, UNITED STATES OF AMERICA

## Abstract

The integration of single-cell multi-omics data provides a powerful approach for understanding the complex interplay between different molecular modalities, such as RNA expression, chromatin accessibility and protein abundance, measured through assays like scRNA-seq, scATAC-seq and CITE-seq, at single-cell resolution. However, most existing single-cell technologies focus on individual modalities, limiting a comprehensive understanding of their interconnections. Integrating such diverse and often unpaired datasets remains a challenging task due to unknown cell correspondences across distinct feature spaces and limited insights into cell-type-specific activities in non-scRNA-seq modalities. In this work, we propose BiCLUM, a Bilateral Contrastive Learning approach for Unpaired single-cell Multi-omics integration, which simultaneously enforces cell-level and feature-level alignment across modalities. BiCLUM first transforms one modality, such as scATAC-seq, into the data space of another modality, such as scRNA-seq, using prior genomic knowledge. It then learns cell and gene embeddings simultaneously through a bilateral contrastive learning framework, incorporating both cell-level and feature-level contrastive losses. Across multiple RNA+ATAC and RNA+protein datasets, BiCLUM consistently outperforms or matches existing integration methods in both visualization and quantitative benchmarks. Importantly, BiCLUM embeddings preserve biologically meaningful regulatory relationships between chromatin accessibility and gene expression, as evidenced by significantly higher gene–peak correlations than random controls. Downstream analyses further demonstrate that BiCLUM-derived embeddings facilitate transcription factor activity inference, identification of cell-type-specific marker genes, functional enrichment, and cell–cell interaction mapping. Comprehensive hyperparameter sensitivity and ablation analyses further establish BiCLUM as a robust and interpretable framework that not only achieves effective cross-modal alignment but also retains the underlying regulatory and functional landscape across single-cell modalities.

## Author summary

With the rise of single-cell multi-omics technologies, researchers can now probe multiple molecular layers (e.g., transcriptome and epigenome) within individual cells, enabling a more comprehensive understanding of cellular states and functions. However, integrating unpaired multi-omics data from different modalities poses significant challenges due to batch effects, non-overlapping feature spaces, and the lack of cell-to-cell correspondence. To address these issues, we introduce BiCLUM, a bilateral contrastive learning–based integration framework that innovatively models both cell-level and feature-level dependencies in a unified manner. Instead of relying solely on cell-level matching, BiCLUM constructs mutual nearest neighbor (MNN) pairs at both the cell and feature levels, and employs a bilateral contrastive objective to enforce consistent alignment across modalities. This design enables BiCLUM to capture multi-omic correspondences from two complementary perspectives, enhancing the robustness and biological interpretability of the integration. We evaluate BiCLUM across a diverse set of benchmark datasets and show that it consistently outperforms existing integration methods in terms of cell-type alignment, omics mixing, and downstream trajectory inference. Our work highlights the effectiveness of contrastive learning in resolving modality discrepancies and provides a robust framework for integrative single-cell analysis.

## Introduction

In recent years, single-cell analysis has emerged as a pivotal focus in biomedicine, driven by the development of advanced technologies designed to measure various molecular modalities at the single-cell level. For example, scRNA-seq has been widely adopted to profile gene expression [1–3], while chromatin accessibility [4,5], DNA methylation [6,7], and histone modifications [8] have also been explored at single-cell resolution. However, they often focus on individual modalities, limiting the full understanding of the relationships between them. To address this limitation, multimodal technologies [9–11] have been developed to enable the simultaneous measurement of multiple modalities within the same cells. By integrating these modalities, a more comprehensive understanding of the relationships across various cellular dimensions can be achieved, facilitating the construction of informative representations of cells.

The integration of single-cell multi-omics datasets can be broadly categorized into paired and unpaired scenarios. In the paired scenario, multiple modalities are measured from the same cells. For example, Mowgli [12] is a joint integration approach that is based on integrative Nonnegative Matrix Factorization and Optimal Transport, specifically designed for such datasets. However, despite advancements in technologies capable of simultaneously profiling multiple modalities, conducting multi-omics experiments remains technically challenging and resource-intensive. Although recent multimodal technologies such as 10x Multiome ATAC+RNA, SHARE-seq [13], and ASAP-seq [14] enable simultaneous profiling of multiple molecular layers within the same cell, a substantial number of single-cell multi-omics datasets are still generated from separate experiments involving different cell batches, resulting in unpaired datasets. These unpaired datasets pose significant challenges for integrative analysis across individuals, technologies, and species. The challenges of unpaired integration of multi-omics datasets primarily fall into two aspects. Firstly, different modalities lack common features, and the correspondences between features and cells are unknown. Secondly, compared to scRNA-seq data, the cell-type-specific activity of features in other modalities is less well established, posing additional challenges for effective integration.

Several methods have been developed to address the integration of single-cell multi-omics datasets in unpaired scenarios, which can be broadly classified into two categories. The first category of approaches focuses on aligning cells across different omics modalities using nonlinear manifold alignment techniques. Different methods in this category align the modalities either before embedding learning (e.g., UnionCom [15], Pamona [16]), after embedding learning (e.g., scTopoGAN [17]), or simultaneously during embedding learning (e.g., MMD-MA [18], JointMDS [19], MultiVI [20], scMoMaT [21]). For example, UnionCom [15] aligns cells by matching geometric distance matrices of raw data across modalities, then projects them into a shared low-dimensional space where cells with inferred correspondences exhibit similar expression patterns. scTopoGAN [17] employs topological autoencoders to extract latent representations of each modality separately and utilizes a topology-guided generative adversarial network (GAN) to align these representations in a common feature space. JointMDS [19] integrates multidimensional scaling (MDS) with Wasserstein Procrustes analysis to simultaneously derive low-dimensional embeddings and infer correspondences across modalities. MultiVI [20] is a deep generative model that learns a shared latent representation across multiple omics layers through nonlinear manifold alignment, and an additional alignment loss encourages the latent embeddings of paired cells from different modalities to be close in the latent space. scMoMaT [21] is a matrix tri-factorization-based framework designed for single-cell mosaic integration and multi-modal biomarker detection. It jointly learns shared low-dimensional representations of cells and features across partially overlapping batches and modalities by decomposing each observed matrix into cell-specific, feature-specific, and modality–batch-specific latent factors. These integration methods for unpaired single-cell multi-omics datasets leverage a variety of innovative techniques combining latent embedding learning and manifold alignment to provide meaningful insights into cellular and molecular relationships. However, these manifold alignment-based methods may not fully consider biological relationships between modalities, potentially resulting in outcomes that lack biological interpretability.

The second category of approaches involves converting multi-omics datasets into a unified feature space, often leveraging prior biological knowledge. This common representation enables the integration of diverse datasets by highlighting shared patterns or features across modalities. Most existing methods in this category focus on integrating scRNA-seq and scATAC-seq datasets [22,23] by leveraging prior genomic information to convert ATAC features into RNA feature space. Specifically, these approaches convert scATAC-seq data into gene activity scores, thereby generating shared features (i.e., genes). For example, the Seurat3 method [24] identifies anchors using Canonical Correlation Analysis (CCA) and Mutual Nearest Neighbors (MNN), followed by filtering, scoring, and weighting these anchors to obtain a corrected expression matrix. The LIGER method [22] aligns datasets by identifying shared and dataset-specific factors through integrative non-negative matrix factorization. These two methods employ linear transformations, which may not sufficiently capture the complex topological structures inherent in different modalities. bindSC [25] adopts a different approach by using a transformed gene activity expression matrix as a bridge between multi-omics datasets and applying canonical correlation analysis (CCA) to align datasets at both the feature and cell levels. scCross [26] is another deep generative framework that combines modality-specific VAEs, GAN-based regularization, and mutual nearest neighbor (MNN) anchors to achieve cross-modal alignment and enable cross-modal data generation and in silico perturbations. scCross also integrates gene-set score vectors (gene activity score matrix) as biological priors to enhance alignment quality. Some methods, instead of directly transforming features across modalities, leverage genomic information to establish connections between features. For example, GLUE [27] learns latent embeddings through a guidance graph to link features across modalities and uses an adversarial network to align cells. The guidance graph is a graph-based structure that leverages prior biological knowledge to establish relationships between features across different modalities, facilitating their integration. Similarly, scDART [28] uses prior knowledge to define a linkage matrix between features across modalities and aligns datasets via Maximum Mean Discrepancy (MMD). However, the method requires computing similarity matrices for each modality, which can cause memory issues with large datasets.

Another limitation is that most existing integration approaches are specifically designed for scRNA-seq and scATAC-seq datasets, thereby limiting their applicability to other modalities, such as the alignment of gene expression with (surface) protein expression. In this context, protein features are sparse in the sense that only a limited number of proteins are measured, and the features across modalities exhibit weak linkages, thus constructing an effective guidance graph is particularly challenging.

In this work, we propose BiCLUM, a novel multi-omics integration method that simultaneously enforces cell-level and feature-level alignment across modalities to integrate gene expression (scRNA-seq) with chromatin accessibility (scATAC-seq) or protein expression (CITE-seq). BiCLUM first transforms multi-omics data into a common feature space using prior biological knowledge, such as gene-peak or gene-protein relationships, for integrating gene expression with chromatin accessibility or gene expression with protein expression, respectively. It then learns cell and feature embeddings simultaneously through a bilateral contrastive learning framework, incorporating both cell-level and feature-level contrastive losses. For cell-level alignment, BiCLUM utilizes the Mutual Nearest Neighbors (MNN) method to establish correspondences between cells based on their gene expression levels across different modalities. For feature-level alignment, BiCLUM leverages a one-to-one correspondence between shared features, ensuring consistency across datasets. By utilizing these correspondences, BiCLUM employs bilateral contrastive learning to derive a low-dimensional representation of cells, ensuring that similar cell types are clustered together while maintaining distinct separations for different cell types. BiCLUM is applied to integrate gene expression with chromatin accessibility using four scRNA-ATAC-seq multi-omic datasets and a CITE-seq dataset. While pairing information is available for some datasets, it is only used for quantitative evaluation. Extensive benchmarking results show that BiCLUM outperforms other state-of-the-art approaches, achieving superior quantitative metrics and visualizations. Furthermore, in downstream biological analysis, BiCLUM demonstrates superior or comparable integration performance compared to existing methods, highlighting its ability to preserve biological characteristics and reveal meaningful biological insights. This robustness in practical applications underscores the potential of BiCLUM for multi-omics data integration.

## Results

### Overview

Integration of single-cell multi-omics datasets is crucial for a comprehensive understanding of cellular mechanisms and heterogeneity. However, integrating these datasets, especially in unpaired scenarios, poses significant challenges due to the lack of direct correspondence between cells from different modalities. Existing methods address this issue by aligning cells via nonlinear manifold alignment or transforming datasets into a common feature space based on prior knowledge. Despite these advances, integrating datasets across different batches and experiments remains a complex task.

**Fig 1:**
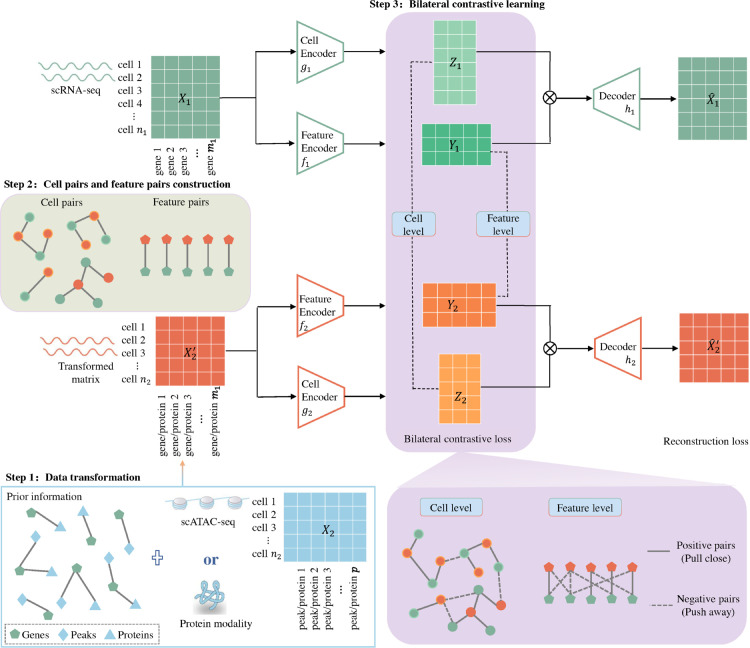
Overview of BiCLUM for unpaired integration of scRNA-seq (*X*_1_) and other modalities such as scATAC-seq or protein data (*X*_2_). The method involves three key steps: (1) step 1: data transformation. Firstly transforming the data from other modalities to obtain a transformed matrix, $X_{2}^{\prime }$. For scATAC-seq data, transforming the modality data into an inferred gene activity score matrix, ensuring that both *X*_1_ and $X_{2}^{\prime }$ share the same set of features. For the protein modality, the $X_{2}^{\prime }$ includes only proteins encoded by some of the genes present in *X*_1_. (2) step 2: cell pairs and feature pairs construction. The cell level pairs and the feature level pairs across modalities can be constructed based on *X*_1_ and $X_{2}^{\prime }$. (3) step 3: bilateral contrastive learning. Separate encoders are employed for learning the cell-level ($g_{v},v=1,2$) representations and feature-level ($f_{v},v=1,2$) representations for each modality. The bilateral contrastive learning is to enforce alignment at both the cell and feature levels across modalities. Finally, the latent cell and feature embeddings are passed through modality-specific decoders ($h_{v}$, v=1,2) to reconstruct the original features from both modalities.

In this study, we introduce BiCLUM (Bilateral Contrastive Learning for unpaired Multi-omics), an innovative approach for unpaired multimodal data integration based on contrastive learning. BiCLUM extends MNN-based alignment (e.g. scCross) to both the cell and feature domains and integrates them through a bilateral contrastive objective, offering a novel dual-perspective mechanism for cross-modal correspondence. As depicted in Fig 1, before learning the latent representation of cells, we first establish a connection between cross-modal data at both the cell and feature levels. Specifically, by first performing a feature space transformation, BiCLUM transforms features from various modalities into a gene activity score matrix or selects features relevant to scRNA-seq data. This ensures that features between different datasets are comparable, thus forming a common basis for integration. A mutual nearest neighbor (MNN) algorithm is then used to establish correspondence between cells from different modalities based on gene expression levels. Leveraging correspondences identified at the cell and feature levels, BiCLUM applies bilateral contrastive learning to learn low-dimensional representations of cells, enhancing the capture of biological relationships and improving integration accuracy.

By applying BiCLUM to five real-world multi-omics datasets (including both paired and unpaired scRNA-seq and scATAC-seq data, as well as paired CITE-seq data measuring gene expression and surface protein levels), and comparing it against 16 state-of-the-art methods, we demonstrate that BiCLUM effectively aligns heterogeneous omics data into a unified low-dimensional representation. Although some datasets were originally paired, we treated them as unpaired during training, using the pairing information only for quantitative evaluation. In downstream biological analyses, BiCLUM preserves the biological integrity of the data, underscoring its utility in uncovering meaningful biological insights and its robustness for practical applications.

### BiCLUM achieves robust multi-omics PBMC integration with well cell type preservation

We first evaluated BiCLUM on the integration of scRNA-seq and scATAC-seq data using two PBMC datasets: the paired PBMC dataset, which contains simultaneous scRNA-seq and scATAC-seq measurements, and the unpaired PBMC dataset, where the two modalities were obtained separately. We compared our alignment results with the raw data and those obtained from 16 state-of-the-art integration methods through both visualization and quantitative evaluation of the integration results.

#### Paired PBMC data analysis.

**Fig 2:**
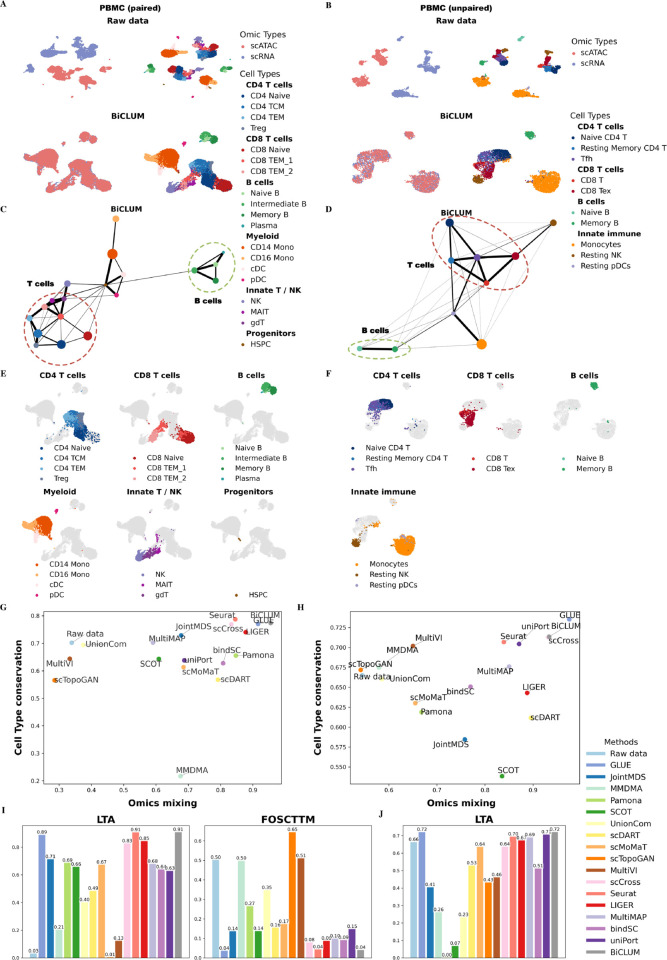
Integrated results of paired PBMC and unpaired PBMC datasets. (A–B) UMAP visualizations of the embeddings for the raw data and BiCLUM method with cells colored by omic types and cell types for the two datasets, respectively. (C–D) PAGA trajectory visualizations of the integrated embeddings from BiCLUM for the two datasets. Cell types from the same lineage are grouped and highlighted with dotted ovals for clarity. (E-F) Highlighted lineage-specific UMAP visualizations of the BiCLUM embeddings for the two datasets. For each panel, a major cell compartment is emphasized while all other cells are displayed in light gray, accompanied by a focused legend and panel title. (G–H) Comparison of omics mixing and biological conservation metrics across different multi-omics integration methods for the paired and unpaired PBMC datasets, respectively. (I-J)LTA and FOSCTTM scores across different integration methods for the paired (I) and unpaired (J) datasets.

In Fig 2A, we present UMAP visualizations of the raw, unintegrated data and the embeddings generated by BiCLUM. To further facilitate interpretation of lineage organization and enhance visual clarity for specific cell compartments, we additionally provide highlighted lineage-specific UMAP visualizations of BiCLUM embedding in Fig 2E. In these panels, cells belonging to a given lineage are emphasized, whereas all other cells are displayed in light gray, allowing clearer inspection of lineage continuity and substructure. Visualizations of embeddings produced by the other compared methods are provided in S1 Fig. The UMAP results highlight the integration performance across methods. The raw data display clear modality-specific separation, with scATAC-seq and scRNA-seq cells forming distinct, non-overlapping clusters. Methods such as bindSC, LIGER, Pamona, and scDART achieve partial alignment between modalities but still exhibit incomplete integration. In contrast, GLUE, Seurat, and BiCLUM show substantially improved alignment, yielding well-mixed scATAC-seq and scRNA-seq cells while maintaining clear, biologically meaningful clustering by cell type, as also illustrated in the lineage-highlighted views in Fig 2E.

Fig 2C displays PAGA graphs of the BiCLUM-integrated results, which effectively highlight the functional and developmental relationships among cell types. BiCLUM captures key differentiation trajectories, such as the differentiation of CD4+ and CD8+ Naive cells into Treg, TCM, and TEM cells, as well as the differentiation of naive B cells into intermediate B cells, memory B cells, and plasma cells. These observations are in alignment with existing literature [29–31], thereby validating the biological relevance of BiCLUM’s integration and trajectory inference. Additionally, PAGA graphs for comparison methods are shown in S2 Fig. Notably, with the exception of the MMDMA method, most methods reveal relatively clear trajectory paths. However, methods such as GLUE, MultiMAP, and UnionCom fail to reflect the role of HSPCs as the origin of all blood cell types, displaying few or weak edges emanating from HSPCs. Furthermore, methods such as bindSC, Seurat, and SCOT misplace plasma cells near HSPCs, leading to biologically implausible connections with multiple cell types, which contradicts their distinct lineage origins. The PAGA graph analysis, coupled with the UMAP clustering shown in Fig 2A, underscore BiCLUM’s superior performance in integrating multi-omics data and producing biologically meaningful differentiation trajectories.

Fig 2G further quantifies the integration outcomes, while Seurat attains the highest cell type conservation, its omics mixing score is lower than that of BiCLUM. In contrast, BiCLUM achieves a balanced performance between biological conservation and modality alignment. In addition, Fig 2I shows that BiCLUM exhibits the highest LTA and the lowest FOSCTTM values, reflecting its superior ability to achieve both accurate cross-omics alignment and well-preserved biological structure.

#### Unpaired PBMC data analysis.

Unlike the paired PBMC data, the transformed gene activity score matrix in the the PBMC (unpaired) dataset was generated using the MAESTRO method, as described in the original study [32]. As shown in Fig 2B and S3 Fig, UMAP visualizations reveal that GLUE, uniPort, and BiCLUM achieve the most effective integration results, successfully aligning scRNA-seq and scATAC-seq modalities while preserving clear cell type boundaries. To further illustrate lineage organization, we also provide highlighted lineage-specific UMAP visualizations of the BiCLUM embedding in Fig 2F. These panels show that cells belonging to the same lineage are positioned closely in the embedding space, reflecting both cross-modality alignment and biologically coherent structure.

In Fig 2D, BiCLUM’s integrated embeddings reveal coherent differentiation trajectories within major immune lineages. Within the CD8 T cell compartment, a clear transition from CD8 T cells to CD8 Tex (exhausted T cells) is observed, consistent with previous findings that effector CD8 T cells gradually acquire an exhausted phenotype under chronic infection or tumor microenvironment conditions [33]. For CD4 T cells, BiCLUM captures biologically meaningful trajectories of immune activation, in which naïve CD4 T cells differentiate into T follicular helper (Tfh) cells, followed by the emergence of resting memory Tfh cells that contribute to long-term immune memory [34]. In the B cell lineage, the inferred path from naïve B cells to memory B cells mirrors the canonical germinal center maturation process. Interestingly, BiCLUM also detects potential cross-lineage interactions between Tfh and CD8 T cells. While Tfh functions are traditionally attributed to CD4 Tfh cells, recent evidence has identified a distinct CXCR5^+^PD-1^+^ CD8 T cell subset sharing transcriptional features with CD4 Tfh cells and capable of supporting B cell activation and antibody production [35]. Together, these results demonstrate that BiCLUM faithfully reconstructs known immune differentiation hierarchies and reveals nuanced regulatory relationships between lymphocyte subtypes. A PAGA analysis of the comparison methods is provided in S4 Fig. Notably, several integration approaches such as GLUE, scMoMaT, MultiVI, Seurat, LIGER, MultiMAP and uniPort, also reveal relatively clear trajectory paths. The similarity in lineage organization across these methods indicates that the trajectories inferred by BiCLUM are broadly consistent with lineage patterns recovered by other established integration frameworks.

The quantitative metrics of omics mixing and cell type conservation in Fig 2H, together with cell type accuracy in Fig 2J, highlight the superior performance of BiCLUM, which achieves comparable transfer accuracy to GLUE while effectively preserving cell type identities across modalities, outperforming other integration methods.

### BiCLUM excels in integrating multi-omics Kidney data and achieves well-mixed omics and distinct separation of cell types

**Fig 3:**
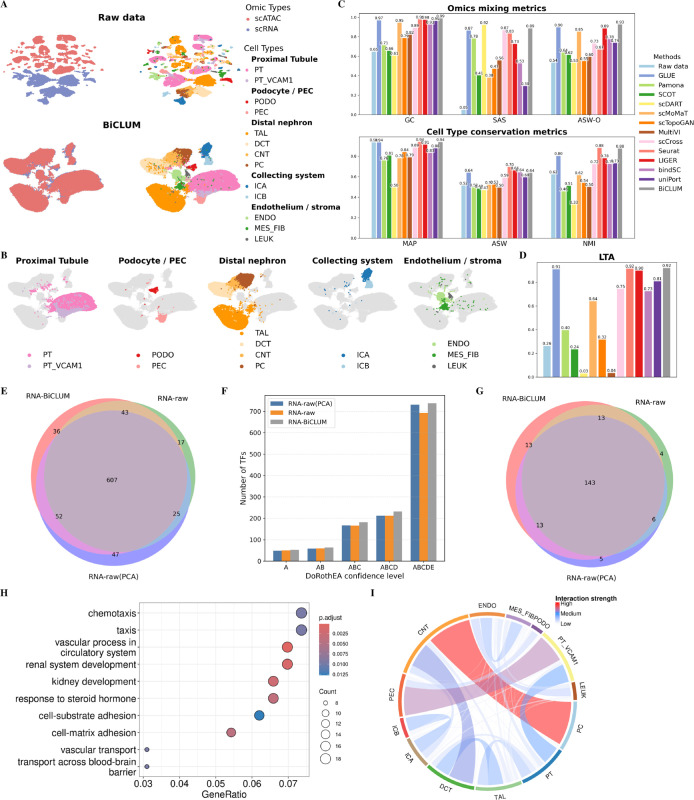
Integrated analysis of Kidney data. (A) UMAP visualizations of cell embeddings obtained from raw data and BiCLUM, colored by omic types and cell types, respectively. (B) Highlighted lineage-specific UMAP visualizations of the BiCLUM emdedding. For each panel, a major cell compartment is emphasized while all other cells are shown in light gray, accompanied by a focused legend and panel title. (C) Comparison of multi-omics integration methods based on omics mixing (GC, SAS, ASW-O) and biological conservation (MAP, ASW, NMI) metrics. (D) LTA scores of different integration methods. (E) Venn diagram comparing transcription factors (TFs) identified from BiCLUM-derived gene embeddings (RNA-BiCLUM), raw RNA data (RNA-raw) and RNA-raw(PCA) embeddings based on the DoRothEA-based enrichment analysis. (F) Cumulative numbers of TFs identified by RNA-BiCLUM, RNA-raw, and RNA-raw(PCA) embeddings at increasingly inclusive DoRothEA confidence levels (A, AB, ABC, ABCD, ABCDE). (G) Venn diagram comparing subsets of TFs identified by each gene embedding that have at least one known target annotated in DoRothEA with confidence levels A–C. (H) GO enrichment analysis of cell type–specific marker genes identified from RNA-BiCLUM gene embeddings. (I) Inference of potential cell–cell interactions from RNA-BiCLUM cell embeddings.

We further evaluated the performance of BiCLUM on the Kidney dataset. As shown in Fig 3A and S5 Fig, UMAP plots illustrate the integration results for various methods. In the raw data, omics datasets are not well mixed, with most cell types nearly separated, except for certain cell types like CNT and DCT, which are not clearly clustered. BiCLUM demonstrates superior performance by not only achieving effective mixing of the two omics datasets but also ensuring that cells of the same type are well-clustered. In comparison, other methods show varying levels of integration quality. While some methods, such as scDART, GLUE, Seurat, and bindSC, successfully mix scRNA-seq and scATAC-seq data, their cell type separation remains suboptimal. For instance, bindSC exhibits significant mixing of distinct cell types. scDART struggles to separate certain cell types with fewer cells. GLUE fails to distinguish between PODO cells and LEUK cells, as well as between ICA and ICB cells. Seurat encounters difficulties in separating LEUK and ENDO cells, leading to partial overlap between these two types. The lineage-specific UMAP visualization of the BiCLUM embedding in Fig 3B highlights that cells of the same lineage are closely positioned in the embedding space, reflecting BiCLUM’s ability to achieve cross-modal alignment while preserving biologically coherent structure.

Fig 3C quantifies the performance of different methods across individual metrics associated with omics mixing and cell type conservation. Notably, BiCLUM achieves the highest scores for GC and ASW-O, indicating superior omics integration. In terms of cell type conservation, it attains the highest MAP and NMI values, while the remaining metrics rank second best, further demonstrating its ability to preserve distinct cellular identities. Moreover, Fig 3D demonstrates that BiCLUM achieves the highest LTA among all methods, further emphasizing its effectiveness in integrating unpaired multi-omic data.

#### Transcription factor activity inference and benchmarking.

Beyond cell embeddings used for clustering and trajectory analysis, the gene embeddings learned by BiCLUM can also be leveraged to characterize transcriptional regulatory programs. In this analysis, we focused on gene embeddings obtained from the RNA-only branch of BiCLUM (RNA-BiCLUM). Each gene was represented by its embedding vector, and a gene–gene similarity network was constructed based on cosine similarity in the embedding space. For each gene, edges were drawn to its *k* nearest neighbors (*k* = 5 by default), resulting in a weighted, undirected gene–gene similarity graph. Gene modules were then identified using greedy modularity optimization as implemented in NetworkX. Each module thus represents a group of genes with highly similar embedding patterns, potentially corresponding to co-regulated or functionally related gene sets.

To associate transcriptional regulators with each gene module, we performed transcription factor (TF) enrichment analysis using the DoRothEA database, a curated resource of TF–target interactions annotated with graded confidence levels (A–E). For each module, enrichment was evaluated by testing whether the known target genes of a TF were significantly overrepresented among the genes assigned to that module relative to a genome-wide background. Statistical significance was assessed using a one-sided hypergeometric test, and the resulting *p* values were adjusted for multiple hypothesis testing using the Benjamini–Hochberg FDR [36] procedure. TFs with FDR-adjusted *p* values below 0.05 in at least one module were retained, and the union of TFs across all modules constituted the final TF set for each method.

For comparison, we applied the same analysis pipeline to raw gene-by-cell RNA expression data (RNA-raw). To further disentangle the effect of dimensionality reduction from that of multimodal representation learning, we additionally applied the same pipeline to gene embeddings derived from raw RNA expression after principal component analysis (RNA-raw(PCA)). For this PCA baseline, PCA was performed on the cell-by-gene RNA expression matrix, and gene embeddings were defined as the gene loading vectors in the PCA space.

We first compared the TFs identified from RNA-BiCLUM gene embeddings with those obtained from RNA-raw and RNA-raw(PCA) using the same enrichment framework. As shown in Fig 3E, the numbers of identified TFs for RNA-BiCLUM, RNA-raw, and RNA-raw(PCA) are 738, 692, and 731, respectively. The three TF sets exhibit substantial overlap, with 607 TFs shared by all three embeddings, indicating that a large fraction of regulatory signals captured by RNA-BiCLUM is consistent with those supported by the original expression data.

To assess whether different embedding strategies influence the distribution of annotation support among the identified TFs, we next compared the number of TFs across cumulative DoRothEA confidence levels (A, AB, ABC, ABCD, and ABCDE). As shown in Fig 3F, although RNA-raw(PCA) yielded a comparable total number of TFs, these TFs were predominantly associated with lower-confidence DoRothEA annotations, and the number of TFs supported by higher-confidence evidence remained similar to that obtained from raw RNA expression. In contrast, RNA-BiCLUM tended to identify more TFs across cumulative confidence levels and exhibited a relative enrichment of TFs whose regulatory annotations are supported by medium- to high-confidence DoRothEA evidence. This pattern suggests that the representation learned by RNA-BiCLUM emphasizes more coherent and consistently annotated regulatory signals.

We further examined TFs uniquely identified by each method. RNA-BiCLUM, RNA-raw, and RNA-raw(PCA) uniquely identified 36, 17, and 47 TFs, respectively. Among these unique TFs, 13 of 36 for RNA-BiCLUM, 4 of 17 for RNA-raw, and 5 of 47 for RNA-raw(PCA) have known target genes annotated in DoRothEA with confidence levels A–C (Fig 3G). The higher proportion of TFs with high-confidence regulatory annotations among those uniquely identified by RNA-BiCLUM further supports its ability to capture biologically meaningful regulatory programs beyond those recovered from raw expression or linear dimensionality reduction.

#### Gene marker identification and enrichment analysis.

Gene embeddings obtained from the RNA-BiCLUM model were further utilized to identify cell type–specific marker genes. For each gene, we calculated its similarity with all cell embeddings to construct a gene–cell similarity matrix. For each cell type, the top 50 genes with the highest similarity scores were selected as candidate markers. Marker genes across all cell types were then aggregated to form a comprehensive gene set for downstream analyses.

To evaluate the biological relevance of these marker genes, we performed Gene Ontology (GO) enrichment analysis, the top 10 most significantly enriched GO biological-process terms (*adj*.*p* < 0.05) are displayed in Fig 3H. The analysis revealed significant enrichment of biological processes closely related to kidney function and development. Notably, key terms such as renal system development, kidney development, and vascular process in circulatory system were among the most significantly enriched, indicating that our identified marker genes are tightly linked to essential physiological processes of the kidney. In addition, enrichment of terms such as cell–substrate adhesion, cell–matrix adhesion, and vascular transport suggests active participation in structural organization and transport mechanisms within the renal microenvironment. The enrichment of response to steroid hormone further supports the hormonal responsiveness of renal cells, consistent with known kidney biology. Overall, the GO enrichment results validate that the identified marker genes are biologically meaningful and reflect key processes underlying kidney development, structure, and function.

#### Inference of potential cell–cell interactions from RNA-BiCLUM cell embeddings.

To characterize potential interactions between kidney cell types, we constructed a cell–cell similarity network based on RNA-BiCLUM embedding. For each cell, we identified its k-nearest neighbors (*k* = 100) using cosine similarity and built a weighted undirected graph, which was then aggregated at the cell-type level and row-normalized to quantify the relative interaction propensity between cell types. To highlight dominant interactions, only the upper triangle of the symmetric matrix was retained, and edges with weights <0.005 were masked, preserving approximately the top 33% strongest cross-type connections (Fig 3I).

The chord diagram depicts the cell–cell interaction network within the renal cortex, stratified by three levels of interaction strength. Strongest interactions (darkest colors) occur between proliferating epithelial cells (PC) and connecting tubules (CNT), and between parietal epithelial cells (PEC) and VCAM1+ proximal tubules (PT_VCAM1), likely reflecting epithelial turnover and structural coupling of glomerular filtration units, respectively. Medium-strength interactions are observed between distal convoluted tubules (DCT) and CNT, proximal tubules (PT) and PT_VCAM1, the thick ascending limb (TAL) and DCT, A/B-type intercalated cells (ICA and ICB), as well as ENDO and mesenchymal fibroblasts (MES_FIB), highlighting nephron-segment continuity and functional crosstalk in the vascular-interstitial microenvironment. Weakest interactions correspond to background similarities without clear anatomical relationships.

Consistent with these proximity patterns, PAGA-based trajectory analysis (S6 Fig) highlights coherent functional and developmental relationships among major cell types, including glomerular cells (PT, PEC, PODO) [37,38], distal nephron segments (TAL, DCT, CNT, PC) [39], and intercalated cells (ICA, ICB) [40]. While PAGA captures developmental trajectories, the chord diagram reflects microanatomical adjacency. Together, these analyses demonstrate that BiCLUM simultaneously encodes both lineage trajectories and spatial proximity, providing biologically consistent and interpretable representations of kidney cellular organization.

### BiCLUM achieves superior quantitative metrics for the integration of scRNA and scATAC data in BMMC data

To further evaluate the performance of BiCLUM, we constructed two datasets from a paired multi-omics experiment containing scRNA-seq and scATAC-seq modalities: paired BMMC and unpaired BMMC. The paired BMMC dataset includes both modalities from the same batch (s1d1), while the unpaired BMMC dataset combines the RNA modality from batch s1d1 and the ATAC modality from batch s1d2.

**Fig 4:**
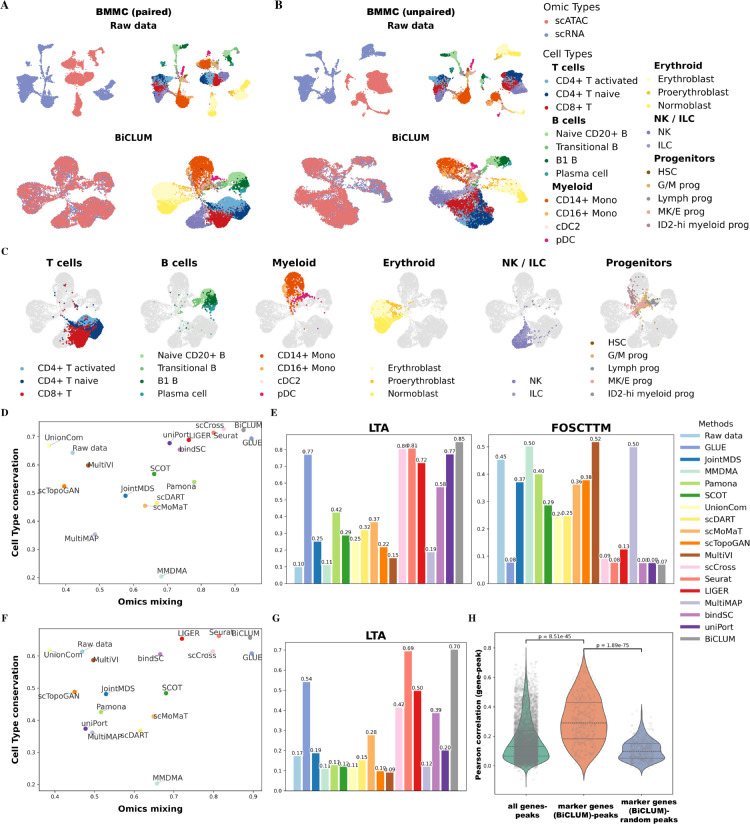
Integrated results of paired BMMC and unpaired BMMC datasets. (A–B) UMAP visualizations of embeddings from raw data and BiCLUM for the two datasets, with cells colored by omics type and cell type. (C) Highlighted lineage-specific UMAP visualizations of the BiCLUM embedding for the paired BMMC dataset. For each panel, a major cell compartment is emphasized while all other cells are displayed in light gray, accompanied by a focused legend and panel title. (D, F) Evaluation of multi-omics integration methods using omics mixing and biological conservation metrics for the paired (D) and unpaired (F) datasets. (E, G) LTA and FOSCTTM scores across different integration methods for the paired (E) and unpaired (G) datasets. (H) Distribution of Pearson correlation coefficients between scATAC-derived gene activity scores and scATAC-seq peak accessibility for the paired BMMC dataset. Three groups are shown: (i) all genes linked to nearby peaks based on genomic proximity; (ii) BiCLUM-identified marker genes paired with their linked peaks; and (iii) the same marker genes paired with randomly selected peaks, *p*-values from two-sample t-tests are indicated.

UMAP visualizations of the integrated embeddings obtained by BiCLUM are shown in Fig 4A and Fig 4B for the paired and unpaired datasets, respectively. The corresponding UMAPs for other methods are presented in S7 and S8 Figs. Overall, GLUE, Seurat, scCross, and BiCLUM effectively integrated the two modalities in both datasets, with cells of the same type clustering together and distinct cell types clearly separated. In contrast, the remaining methods either failed to achieve proper omics mixing or showed poor separation of cell types. To further facilitate the interpretation of lineage organization, Fig 4C presents highlighted lineage-specific UMAP visualizations of the BiCLUM embedding for the paired BMMC dataset. These panels emphasize major cell compartments while showing all other cells in light gray. Collectively, these visualizations demonstrate that BiCLUM achieves both effective cross-modal integration and biologically coherent embedding structures, providing an intuitive representation of cellular organization and lineage relationships.

To further assess the biological relevance of these embeddings, we performed trajectory analysis for the BMMC (paired) dataset using PAGA graphs (S9 Fig). BiCLUM effectively reconstructs key differentiation pathways in hematopoiesis, preserving well-defined transitions from hematopoietic stem cells to progenitors and differentiated cell types. It accurately captures lymphoid and myeloid lineage progressions, erythropoiesis, and dendritic cell development, aligning with known biological hierarchies [41–44]. In contrast, some comparison methods produce disordered graphs that obscure differentiation trajectories, though GLUE, scCross, UnionCom and uniPort successfully capture certain key transitions. These results highlight BiCLUM’s strength in both modality alignment and biological trajectory preservation.

Quantitative results for the paired BMMC and unpaired BMMC datasets are summarized in Fig 4D–4E and Fig 4F–4G, respectively. As shown in Fig 4D and Fig 4F, BiCLUM achieves the best overall integration performance, exhibiting the highest omics mixing and strong cell type conservation, closely following GLUE. In addition, Fig 4E and Fig 4G demonstrate that BiCLUM attains the highest LTA and the lowest FOSCTTM scores, indicating superior cross-omics alignment and improved clustering consistency across cell types.

In the BiCLUM framework, we integrated scRNA-seq data with the gene activity score matrix derived from scATAC-seq data. Since the gene activity matrix bridges chromatin accessibility and gene expression, it is essential to ensure that it preserves biologically meaningful regulatory relationships with the ATAC modality. To assess this, we evaluated whether BiCLUM maintains such regulatory consistency in the paired BMMC dataset. Specifically, we computed Pearson correlations between gene expression (from the scATAC-derived gene activity matrix) and the average accessibility of linked peaks in scATAC-seq. For each gene, the accessibility of all associated peaks, which were identified using the LinkPeaks function in Signac, was aggregated, and the resulting values were correlated with the corresponding gene expression across matched cells. We further repeated this analysis for cell type–specific marker genes identified from BiCLUM gene embeddings, following the same procedure as used for the Kidney dataset. As a control, an equal number of peaks per marker gene were randomly selected to form marker–random peak pairs.

The distribution of correlation coefficients is shown in Fig 4H. From left to right, the three violin plots represent: (i) all genes linked to nearby peaks based on genomic proximity; (ii) BiCLUM-identified marker genes paired with their linked peaks; and (iii) the same marker genes paired with randomly selected peaks. True gene–peak pairs (both all genes and marker genes) show significantly higher correlations than random controls, indicating that BiCLUM preserves intrinsic coupling between chromatin accessibility and gene expression. Notably, marker genes exhibit stronger correlations with their associated peaks (mean = 0.308) compared with all genes (mean = 0.170) or marker–random peak pairs (mean = 0.106). Two-sample t-tests further confirm these differences (Peak–Gene (all genes) vs. Peak–Gene (Marker Gene), $p=8.51\;\times \;10^{-45}$; Peak–Gene (Marker Gene) vs. Marker gene–Random Peaks, $p=1.89\;\times \;10^{-75}$). Collectively, these results demonstrate that BiCLUM effectively captures gene–peak regulatory associations and preserves meaningful chromatin–transcription coupling across modalities.

Notably, although these correlations are computed using the scATAC-derived gene activity matrix and linked peak accessibility rather than the BiCLUM latent embeddings, marker genes were selected based on BiCLUM embedding. Therefore, while this analysis does not directly assess whether the embedding encodes peak–gene associations, the higher correlations observed for marker gene–peak pairs compared with controls indicate that BiCLUM preserves biologically meaningful chromatin–gene relationships during integration.

### BiCLUM reveals consistent, biologically relevant patterns and achieves superior quantitative metrics across BMCITE data

Integration of gene expression and protein abundance is a challenging task as the correlation between mRNA and protein levels can be weak, which is due to factors such as post-transcriptional modifications, differences in degradation rates, and other regulatory mechanisms [45]. Despite these challenges, integrating single-cell RNA and protein data offers significant potential to uncover cellular diversity and functional states. For example, integrating transcriptomic and proteomic data has been used to generate comprehensive maps of aging lung tissue, quantifying activity state changes across cell types [46]. Several computational methods, such as Seurat3, bindSC and so on have applied to the integration of scRNA and protein data.

**Fig 5:**
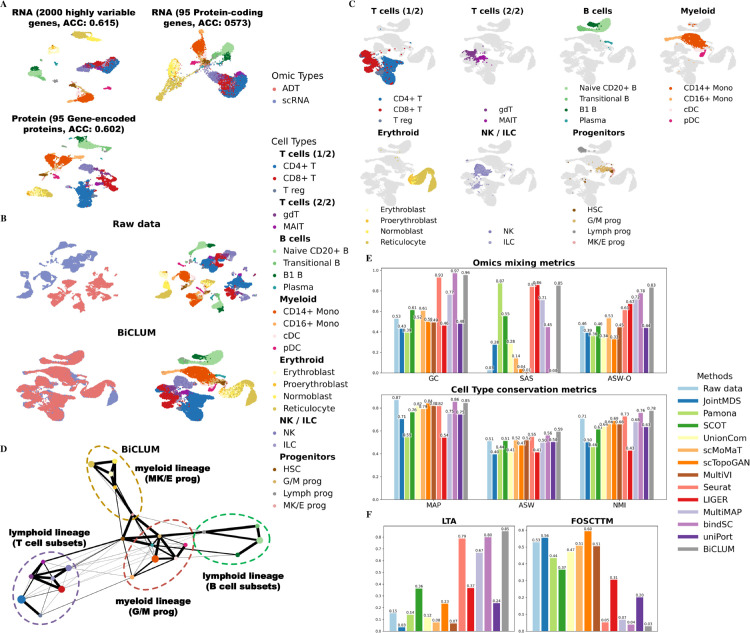
Integrated results of BMCITE data for the two sets, s1d1 and s1d2. (A) UMAP visualization of scRNA-seq data (featuring highly variable and protein-coding genes) and protein data (featuring the corresponding gene-encoded proteins) along with corresponding clustering accuracies. (B) UMAP visualization of the embeddings for the raw data and BiCLUM method, with cells colored based on omics types and cell types, respectively. (C) Highlighted lineage-specific UMAP visualizations of the BiCLUM embedding. For each panel, a major cell compartment is emphasized while all other cells are displayed in light gray, accompanied by a focused legend and panel title. (D) PAGA trajectory visualizations of the integrated embeddings for the BiCLUM method. Cell types from the same lineage are grouped and highlighted with dotted ovals for clarity. (E) All individual metrics belonging to the two evaluation categories, omics mixing (GC, SAS, ASW-O) and biological conservation (MAP, ASW, NMI), in the assessment of multi-omics integration methods. (F) LTA and FOSCTTM values for different integration methods.

**Fig 6:**
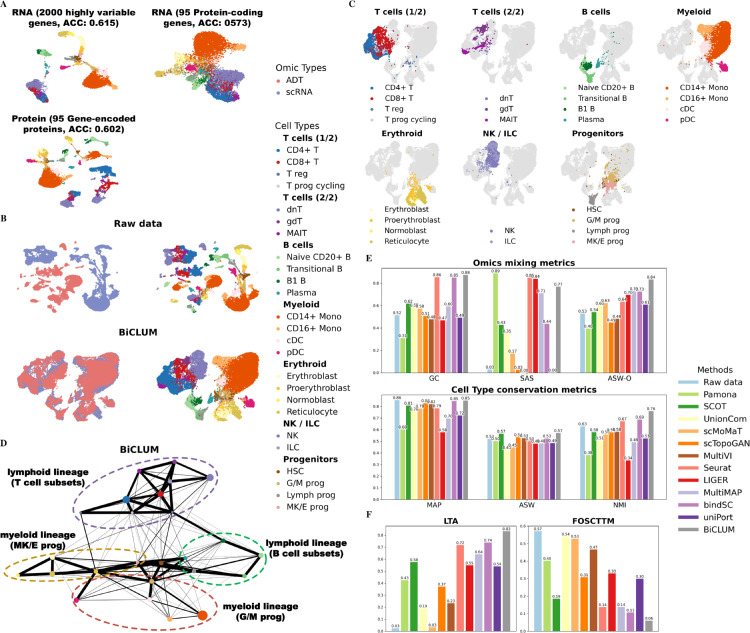
Integrated results of BMCITE data for the two sets, s1d2 and s3d7. (A) UMAP visualization of scRNA-seq data (featuring highly variable and protein-coding genes) and protein data (featuring the corresponding gene-encoded proteins) along with corresponding clustering accuracies. (B) UMAP visualization of the embeddings for the raw data and BiCLUM method, with cells colored based on omics types and cell types, respectively. (C) Highlighted lineage-specific UMAP visualizations of the BiCLUM embedding. For each panel, a major cell compartment is emphasized while all other cells are displayed in light gray, accompanied by a focused legend and panel title. (D) PAGA trajectory visualizations of the integrated embeddings for the BiCLUM method. Cell types from the same lineage are grouped and highlighted with dotted ovals for clarity. (E) All individual metrics belonging to the two evaluation categories, omics mixing (GC, SAS, ASW-O) and biological conservation (MAP, ASW, NMI), in the assessment of multi-omics integration methods. (F) LTA and FOSCTTM values for different integration methods.

We tested our method on a CITE-seq dataset derived from the same cohort of 12 healthy human donors. For evaluation, we selected three subsets of cells, s1d1, s1d2, and s3d7, from the complete dataset. Specifically, we integrated scRNA and ADT for cells from s1d1 and s1d2, with results reported in Fig 5, and for cells from s1d2 and s3d7, with results shown in Fig 6. This design allowed us to evaluate whether our method achieves robust and biologically meaningful integration, ensuring that consistent patterns reflecting true biological insights are identified across datasets without being confounded by data-specific effects. Fig 5A and Fig 6A display clustering results for scRNA-seq data (featuring highly variable and protein-coding genes) and protein data (featuring the corresponding gene-encoded proteins). These results demonstrate that while protein data achieve a comparable accuracy value to scRNA data, cells of the same type in the protein dataset are not as tightly clustered. Further analysis revealed 66% and 62% match rate for cells of the same type within the constructed MNN pairs for the two sets respectively. This indicates that the MNN method effectively aligns features across modalities by utilizing local neighborhood information, even in scenarios where global correlations are weak. This observation is consistent with prior studies showing that RNA and protein measurements capture complementary aspects of cellular biology, representing distinct yet interrelated regulatory layers [47,48].

We then reported the UMAP visualizations of integration results for different methods on the two constructed datasets. In Fig 5B and Fig 6B, we presented the UMAP visualizations of the raw data alongside the embeddings produced by BiCLUM. The UMAP visualizations of the corresponding embeddings after integration by the comparison methods are shown in S10 Fig and S11 Fig. From these visualizations, it can be seen that BiCLUM effectively integrates cells from the two modalities, achieving well-mixed embedding with clear separation between most cell types. In contrast, the compared methods demonstrate varying levels of integration but exhibit notable limitations. For instance, while bindSC and Seurat achieve reasonable integration of the two modalities, their performance falls short in certain aspects, such as the inadequate alignment of reticulocyte cell types across modalities. Other methods either fail to fully integrate the two data modalities or struggle to maintain clear separation between cell types, resulting in overlapping and poorly defined clusters. To facilitate the analysis of lineage organization, highlighted lineage-specific UMAP visualizations of the BiCLUM embeddings are provided in Fig 5C and Fig 6C. In both subfigures, cells belonging to the same lineage occupy contiguous regions in the embedding space, supporting that BiCLUM simultaneously preserves biologically meaningful lineage structure and achieves effective cross-modal integration.

Furthermore, Fig 5D and Fig 6D illustrate the PAGA graphs for both datasets, revealing consistent and biologically relevant differentiation patterns. BiCLUM accurately reconstructs hematopoietic trajectories, capturing well-defined transitions from granulocyte-monocyte progenitors (G/M Progs), megakaryocyte-erythroid progenitors (MK/E Progs), and lymphoid progenitors (Lymph Progs). The central positioning of HSCs and the structured progression of B cells, T cells, monocytes, and erythroid cells align with established hematopoietic hierarchies [49–52], demonstrating the robustness of BiCLUM’s integration.

For both datasets, we further assessed the integration performance of different methods using multiple quantitative metrics evaluating omics mixing and cell type preservation (Fig 5E and Fig 6E). BiCLUM consistently ranked first or second across nearly all metrics, demonstrating its ability to achieve a well-balanced integration that preserves both omics consistency and biological identity. Furthermore, as shown in Fig 5F and Fig 6F, BiCLUM achieved the highest LTA among all methods while maintaining competitive FOSCTTM scores. These results underscore BiCLUM’s effectiveness in integrating multimodal data, ensuring both accurate data alignment and biologically meaningful structure retention.

By evaluating the integration results across multiple subsets of cells, we have demonstrated that the patterns observed are not only robust but also reproducible, underscoring the method’s capacity to generalize across different datasets. This consistency further affirms BiCLUM’s ability to capture true biological relationships.

### Hyperparameter sensitivity and ablation protocol

**Fig 7:**
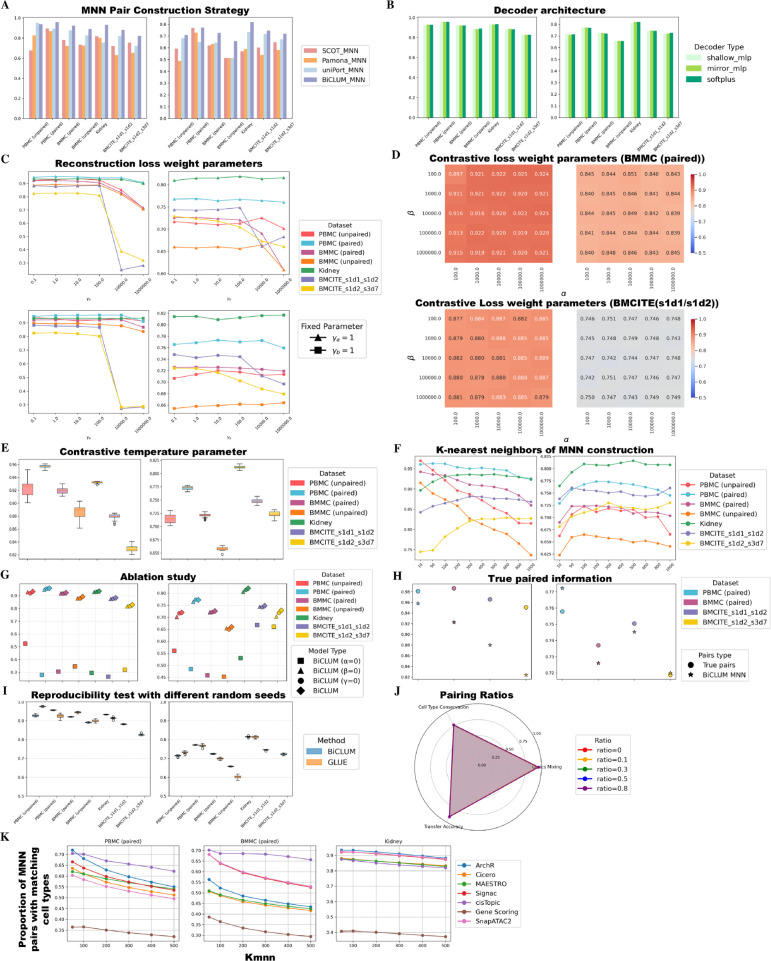
Hyperparameter sensitivity and ablation analysis of the BiCLUM model. Panels (A–I) each consist of two subpanels reporting omics mixing (left) and cell type conservation (right) under different experimental settings: (A) MNN pairs constructed using SCOT, Pamona, uniPort, and BiCLUM. (B) Different decoder architectures. (C) Varying weights of reconstruction losses. (D) Varying weights of contrastive losses. (E) Different contrastive temperature parameters. (F) Different numbers of nearest neighbors used for MNN pair construction. (G) Ablation of key model components. (H) Model performance using true paired information. (I) Reproducibility under different random seeds. (J) Radar plot comparing omics mixing, cell type conservation, and LTA under different cross-modal pairing ratios. (K) Proportion of MNN pairs with matching cell types under different transformations of the gene activity score matrix.

To assess the robustness and reproducibility of BiCLUM, we performed systematic evaluations across its key hyperparameters and architectural components. Integration performance was assessed using omics mixing and cell type conservation metrics, as summarized in Fig 7.

- **MNN Pair Construction Strategy.** We compared our MNN pair construction strategy with those derived from cross-modal matching matrices generated by existing methods, including SCOT, Pamona, and uniPort. Specifically, based on the optimal transport matrix *T* obtained from these methods, we defined cross-modal MNN pairs as cell pairs with relatively high mutual transport probabilities. As shown in Fig 7A, our approach achieves higher integration metric values, indicating that the MNN pairs identified by our method are more suitable for cross-modal integration than those generated by alternative strategies. Furthermore, when compared with the overall integration performance under the SCOT, Pamona, and uniPort frameworks in previous figures, our BiCLUM model exhibits superior cross-modal alignment, highlighting the effectiveness of the proposed contrastive learning design.
- **Varying decoder architecture** In our model, the baseline decoder reconstructs the expression matrix using a softplus activation applied to the bilinear product of cell and feature embeddings. To test whether more complex decoders improve performance, we compared this with two alternatives: shallow MLP decoder and mirror MLP decoder.
  Shallow MLP decoder: applies a single hidden-layer MLP with ReLU and dropout to both embeddings before reconstruction, introducing moderate nonlinearity.
  Mirror MLP decoder: mirrors the encoder architecture in depth and hidden dimensions, applying separate MLPs to the embeddings with the same structure as the corresponding encoder.
  As shown in Fig 7B, the simple softplus decoder achieves comparable or even superior reconstruction and downstream performance. This indicates that adding decoder complexity provides limited benefit while increasing model size and training cost.
- **Varying weights of losses** We evaluated the sensitivity of model performance to the reconstruction loss weighting parameters ($\gamma_{a}$ and $\gamma_{b}$) and the contrastive loss weighting parameters (*α* and *β*). Specifically, for the reconstruction loss, we considered two scenarios: varying $\gamma_{a}$ while keeping $\gamma_{b}=1$, and varying $\gamma_{b}$ while keeping $\gamma_{a}=1$. For the contrastive loss, *α* and *β* were varied across values of 10^2^,10^3^,10^4^,10^5^, and 10^6^.
  As shown in Fig 7C, the evaluation across different datasets indicates that setting $\gamma_{a}$ and $\gamma_{b}$ to comparable magnitudes results in more stable and balanced integration. Similarly, the results for the contrastive loss parameters, reported for the BMMC (paired) and BMCITE_s1d1_s1d2 datasets in Fig 7D, show that choosing similar and relatively large values for *α* and *β* leads to robust and consistent integration outcomes.
- **Varying Contrastive Temperature Parameters.** We evaluated the sensitivity of model performance to the contrastive temperature parameters on different datasets. Specifically, we varied $\tau_{f}=[0.1,0.5,0.9,10,15,50,100]$ while keeping $\tau_{c}=0.5$, and varied $\tau_{c}=[0.1,0.5,0.9,10,15,50,100]$ while keeping $\tau_{f}=0.5$. The evaluation results are summarized as boxplots in Fig 7E. Overall, the relatively tight distribution of the boxplots indicates that the model is robust to the choice of temperature parameters.
- **Varying Number of Nearest Neighbors for MNN Pair Construction.** We further evaluated how the number of nearest neighbors (*k*_*mnn*_) used for MNN pair construction affects integration performance. Specifically, *k*_*mnn*_ was varied across [10,50,100,200,300,400,500,600,800,1000].
  As shown in Fig 7F, integration performance remains consistently high for RNA+ATAC integration when *k*_*mnn*_ is set between 50 and 200, corresponding to approximately 0.1%–5% of the total number of cells in the smaller modality. For RNA+protein integration, higher values of *k*_*mnn*_ (300–1000) yield better performance, suggesting that RNA+protein integration generally requires more nearest neighbors compared to RNA+ATAC integration.
- **Ablation study of key model components.** We performed an ablation study across different datasets by comparing three scenarios: α=0,β=0,γ=0, and the original model. As shown in Fig 7G, *α* has a stronger impact on integration quality than *β*, indicating that cell-level contrastive learning is more critical for the model. The original model generally achieves the best integration performance, suggesting that all three components of the loss function contribute positively to cross-modal integration.
- **With true paired information.** In previous experiments, pairing information was only used for evaluation and not incorporated into the model. Here, we assessed the effectiveness of our model on datasets with known cell pairings. Specifically, for these datasets, MNN pairs were constructed directly using the true correspondences, treating each paired sample as an MNN pair. As shown in Fig 7H, incorporating the true pairing information improves cross-modal integration performance. Importantly, even without using the pairing information, our method achieves comparable integration quality, demonstrating that BiCLUM effectively captures cross-modal relationships and performs robustly under both paired and unpaired settings.
- **Varying Seed for Reproducibility.** To evaluate the robustness of our method to random initialization, we ran experiments with 15 different random seeds across multiple datasets and assessed performance using standard evaluation metrics. We also compared BiCLUM with GLUE under the same seeds. As shown in Fig 7I, BiCLUM exhibits low variability and high consistency across runs, demonstrating its robustness. Furthermore, it achieves comparable or superior integration performance relative to GLUE on most datasets.
- **Varying Pairing Ratios.** To assess the impact of different proportions of matched cells on integration performance, we randomly selected 9,000 paired cells from the two modalities of the paired PBMC dataset and constructed five datasets with matching ratios p=0,0.1,0.3,0.5,0.8. For each dataset, a proportion *p* of the 9,000 pairs was retained as the matched subset, present in both modalities. The remaining unmatched pairs were randomly split into two subsets, each retaining only one modality. For example, at a matching ratio of 0.1, 900 pairs were retained as matched, while the remaining 8,100 pairs were split equally, with 4,050 cells retaining only modality 1 and 4,050 only modality 2. Consequently, each modality contained 4,950 cells, of which only 900 had a corresponding cell in the other modality.
  The evaluation results are shown in Fig 7J. Radar plots of three metrics (omics mixing, cell type conservation, and transfer accuracy) demonstrate that BiCLUM is robust across varying pairing ratios and maintains strong integration performance under different levels of paired modalities.
- **Impact of Gene Activity Score Matrix Transformations.** Since BiCLUM integrates scRNA-seq and scATAC-seq data based on a transformed gene activity score matrix, we evaluated how different transformation methods affect integration quality (Fig 7K). Specifically, we calculated the proportion of MNN pairs with matching cell types.
  The results indicate that ArchR, Signac and cisTopic are the most stable methods, consistently producing high-quality MNN pairs. In contrast, SnapATAC2 generally perform well but occasionally show reduced performance, while Cicero, Gene Scoring, and MAESTRO exhibit relatively lower overall performance. Based on these findings, we recommend using ArchR, Signac or cisTopic for gene activity score matrix transformation when applying BiCLUM to achieve optimal integration results.
- **Computational Complexity and Resource Requirements.** We evaluated the computational demand of BiCLUM across multiple datasets on a workstation with a 24-core CPU (3.4 GHz) and an NVIDIA RTX 3090 GPU (24 GB), summarized in S1 Table. Training time varied with dataset size, ranging from 402±68 s for PBMC (unpaired) to 11,409±1,969 s for Kidney. CPU usage was generally moderate, between 5.7% and 21.1%, while RAM usage scaled with dataset size (15.2%–42.6%). GPU load and memory consumption also varied: small datasets (e.g., PBMC unpaired, BMCITE) used <40% GPU and ∼ 0.5–1.5 GB memory, whereas larger datasets (Kidney, BMMC unpaired) required higher load (27%–50%) and ∼ 1.35–1.76 GB memory. GPU power consumption ranged from ∼ 30 W to 199 W. Overall, BiCLUM is computationally efficient for small- to medium-sized datasets, while larger datasets demand more GPU and RAM resources. Per-epoch complexity scales approximately as O(N·F), where *N* is the number of cells and *F* the number of features.

## Discussion and conclusion

In summary, we developed BiCLUM, a bilaterally contrastive learning framework that extends existing MNN-guided integration paradigms by aligning both cells and features across modalities. Unlike previous methods such as scCross, which primarily rely on cell-level MNN anchors and adopt a VAE–GAN architecture with gene-set priors to guide integration, BiCLUM constructs MNN pairs at both the cell and feature levels and employs a bilateral contrastive objective. This design directly minimizes distances between paired embeddings while maximizing separation from non-paired samples, leading to more discriminative and stable latent representations compared to adversarial training–based approaches.

Comprehensive evaluations on multiple scRNA–scATAC and CITE-seq datasets demonstrate that BiCLUM achieves superior performance in both visualization and quantitative metrics relative to existing methods. It not only produces well-mixed embeddings across modalities but also better preserves biologically meaningful relationships, as reflected in consistent PAGA graphs and improved cell type conservation scores. Direct empirical comparisons further confirm that BiCLUM exhibits stronger cross-modal consistency and enhanced feature-level interpretability than scCross and other recent models.

In practice, we observed that preprocessing pipelines such as ArchR and Signac yield higher-quality gene activity score matrices and more reliable MNN pairs, thereby enhancing integration performance. We therefore recommend using these preprocessing methods when applying BiCLUM to multi-omic datasets.

Despite its promising performance, BiCLUM still has several limitations and opportunities for future improvement. Currently, BiCLUM assumes a straightforward one-to-one correspondence between features across modalities, such as gene-to-gene or gene-to-protein mappings. While effective for many datasets, this assumption may be insufficient for more complex regulatory architectures, where co-expressed or indirectly related genes, combinatorial regulation, or enhancer–gene interactions play a critical role. Future extensions could adopt many-to-many or graph-based linking strategies, similar to approaches used in GLUE, to better capture such intricate relationships and enhance the interpretability and biological relevance of the feature embeddings.

Moreover, the current implementation focuses primarily on integrating two modalities. The integration of three or more modalities, or modalities with limited prior biological knowledge, introduces additional challenges that necessitate more flexible alignment strategies. Incorporating additional biological constraints, leveraging domain-specific insights, or adopting advanced representation learning techniques, such as graph neural networks, may further improve integration quality and provide deeper insights into multimodal data.

While our study focuses on RNA+ATAC and RNA+protein integration, single-cell multi-omics technologies are rapidly expanding to include additional omic layers at high throughput, such as simultaneous genome and transcriptome sequencing with DEFND-seq [53], hybrid BAG-seq [54], and UDA-seq [55]. These emerging technologies reveal more complex, often non-linear cross-layer dependencies that extend beyond simple one-to-one feature correspondences. In principle, the BiCLUM framework can be adapted to such settings, as it relies on learning shared low-dimensional representations guided by cross-modal correspondences rather than modality-specific assumptions. Nevertheless, methodological extensions will be needed to fully capture richer, more intricate relationships across diverse omic layers. Developing such extensions represents an important direction for future research, with the potential to broaden the applicability of BiCLUM and enhance its utility for integrative single-cell multi-omics analyses.

Overall, BiCLUM provides a principled and effective framework for jointly aligning cells and features across single-cell modalities, enabling accurate cross-modal integration and deeper biological interpretability. Looking ahead, we envision that extending BiCLUM to incorporate graph-based biological priors and accommodate multiple omic layers will further enhance its capacity to uncover complex regulatory mechanisms and advance integrative single-cell biology.

## Materials and methods

### Problem statement

Given two single-cell datasets, $X_{v}=[x_{v,1}^{T},\cdots ,x_{v,n_{v}}^{T}]\in R^{n_{v}\times p_{v}},v=1,2$, where $n_{v}$ and $p_{v}$ represent the number of cells and the dimension of features in the *v*-th modality. Direct integration of *X*_1_ and *X*_2_ is challenging not only due to different cells being assayed but also mismatched feature spaces. To address this, we propose a new method that transforms the datasets from the two modalities into a unified feature space.

We address two integration scenarios within a unified framework: the integration of gene expression with chromatin accessibility and the integration of gene expression with protein expression. We denote the single-cell gene expression matrix as *X*_1_ and the chromatin accessibility or protein expression matrix as *X*_2_.

### BiCLUM model

BiCLUM first transforms *X*_2_ to $X_{2}^{\prime }$ using prior biological knowledge, ensuring that both *X*_1_ and $X_{2}^{\prime }$ lie within a unified feature space. This transformation leverages prior biological knowledge, such as gene-peak or gene-protein associations, to establish corresponding cell and feature pairs across modalities. Subsequently, the method learns their embeddings simultaneously through a bilateral contrastive learning framework that incorporates both cell-level and feature-level alignment. A contrastive learning method minimizes the distances between representations of similar points (positive pairs) and maximizes the distances between representations of different points (negative pairs). In our method, we define two types of positive pairs in a bilateral way: mutual nearest neighbor (MNN) pairs between cells, and feature pairs between the two modalities. By leveraging these positive pairs in our bilateral contrastive learning model, we could simultaneously align similar cell types and features from both modalities, improving the quality of integration.

Specifically, the BiCLUM method mainly involves three steps: (1) data transformation, (2) cell pairs and feature pairs construction, and (3) bilateral contrastive learning. These steps are illustrated in Fig 1.

#### Step 1: Data transformation.

Transforming non-RNA modalities to align with the scRNA-seq feature space forms the basis to construct the positive pairs; for either cell pairs or feature pairs, the transformation requires prior information.

To integrate scRNA-seq and scATAC-seq datasets, we first transform scATAC-seq data into a gene activity score matrix using existing methods. These scores estimate gene expression potential by linking chromatin accessibility to genes through regulatory elements. This results in a matrix sharing the same feature space (genes) as scRNA-seq data, enabling direct gene pairing for integration. Several methods, including ArchR [56], Cicero [57], cisTopic [58], SnapATAC [59], scATAC-pro [32], Signac [60], and SnapATAC2 [61], have been developed to transform scATAC-seq datasets into gene activity score matrices for downstream analysis, such as inferring cell types. These methods leverage chromatin accessibility data to infer gene expression potential, focusing on the accessibility of gene regulatory regions like promoters and enhancers. The primary difference among them lies in the calculation and weighting of gene region accessibility. In our work, we primarily use ArchR [56] and Signac [60] for data transformation. Detailed descriptions of these methods are provided in Sect 1 of the S1 Text.

The transformation from single-cell protein data to scRNA data is more straightforward, as it involves directly matching proteins to their corresponding encoding genes, leveraging their one-to-one correspondence. By utilizing known biological associations, such as gene-protein relationships or interactions within cellular pathways, we ensure that both modalities are integrated in a biologically relevant manner.

After transformation, we then use a structured preprocessing pipeline that includes several key steps: identifying highly variable genes (HVGs), cell normalization, log transformation, z-score normalization with truncated values, and principal component analysis (PCA). Note that the PCA step is applied solely to obtain compact input features for the encoder, thereby reducing noise and computational cost. Importantly, PCA embeddings are not used for constructing MNN pairs, which are directly computed based on the original feature space of each modality. The detailed preprocessing procedure is shown in Sect 2 of the S1 Text.

#### Step 2: Cell pairs and feature pairs construction.

Mutual Nearest Neighbors (MNN) have been proposed [62] and widely used in single-cell RNA-seq integration methods, such as Seurat3 [24] and scDML [63], demonstrating its effectiveness in aligning datasets across modalities. In this study, we construct MNN pairs to integrate different modalities. Cell *i* and cell *j* are considered to form an MNN pair if they are mutually nearest neighbors in their respective modalities. That is, the set of MNN pairs between the two modalities is defined as

$$M_{c}^{+}=\{(i,j)|i\in N_{k}^{1}(x_{2,j}),j\in N_{k}^{2}(x_{1,i})\},$$ (1)

where $x_{v,i}$ represents the *i*-th cell in the *v*-th modality, and $N_{k}^{v}(x)$ represents the *k*-nearest neighbors of *x* in the *v*-th modality under cosine distances between the cells. For scRNA and gene activity score matrices, we first identify highly variable genes (HVGs) within each modality and take their union to define a shared feature space. Both modalities are restricted to this HVG union, and cosine distances between cells are computed in this common space. This ensures that similarities are measured on biologically comparable features across modalities. For scRNA and protein data, we adopt the same principle: cosine distances are computed in the shared feature space defined by gene–protein matched pairs, ensuring comparability between modalities.

Gene–protein relationships were obtained from the UniProtKB/Swiss-Prot database. For each protein measured in the single-cell protein modality, we retrieved its corresponding gene symbol(s) according to the official UniProt mappings. These gene–protein pairs were then used to define feature-level correspondences across modalities in BiCLUM.

For the construction of feature pairs, as described in step 1, we combine the HVGs from both scRNA and gene activity score matrices to form gene pairs. Our method focuses exclusively on one-to-one correspondences between these gene pairs, excluding co-expressed gene pairs. When integrating scRNA with single-cell protein data, the feature pairs are constructed by matching genes with their corresponding encoded proteins. We define these constructed feature pairs as $M_{f}^{+}$.

#### Step 3: Bilateral contrastive learning.

Our bilateral contrastive learning is integrated into autoencoder frameworks, ensuring simultaneous alignment of cells and features within low-dimensional embedding spaces. The data *X*_1_ and $X_{2}^{\prime }$ should be well reconstructed through the autoencoders, and meanwhile, the constructed cell pairs and feature pairs should be similar in the embedding spaces. For simplicity, we will use the notation *X*_2_ in place of $X_{2}^{\prime }$ without causing any ambiguity.

Autoencoders are applied to each modality to generate cell embeddings and feature embeddings. Specifically, for each modality *v*, we use distinct cell encoders $g_{v}$ and feature encoders $f_{v}$ to learn the cell embeddings $Z_{v}=g_{v}(X_{v})\in {\mathbb{R}}^{n_{v}\times d}$ and feature embeddings $Y_{v}=f_{v}(X_{v})\in {\mathbb{R}}^{d\times p}$, where *d* is the latent dimension and *p* is the number of features. The encoder networks, $f_{v}$ and $g_{v}$, are parameterized by neural networks with two hidden layers. $X_{v}$ is then reconstructed by product of the cell and feature embeddings. A softplus function $h_{v}$ is applied to this product for nonlinear transformation, resulting in the final reconstructed matrix ${\hat{X}}_{v}$:

$${\hat{X}}_{v}=h_{v}(Z_{v}Y_{v}).$$ (2)

The reconstructed high-dimensional matrix ${\hat{X}}_{v}$ should closely approximate the original input matrix $X_{v}$. To ensure this, we minimize the reconstruction error by constraining the latent cell and feature embeddings of each modality, using the loss function:

$$L_{rec}=\gamma_{a}{\left\|X_{1}-{\hat{X}}_{1}\right\|}_{2}^{2}+\gamma_{b}{\left\|X_{2}-{\hat{X}}_{2}\right\|}_{2}^{2}=\gamma_{a}{\left\|X_{1}-h_{1}(Z_{1}Y_{1})\right\|}_{2}^{2}+\gamma_{b}{\left\|X_{2}-h_{2}(Z_{2}Y_{2})\right\|}_{2}^{2},$$ (3)

where $\gamma =[\gamma_{a},\gamma_{b}{]}^{T}$, with $\gamma_{a}$ and $\gamma_{b}$ controlling the relative contributions of the two modalities to the reconstruction loss, both are set to 1 by default. This process ensures that the embeddings capture the essential structure and features of the original data, facilitating effective integration across modalities.

Bilateral contrastive learning is introduced to ensure that the embeddings of the constructed cell pairs and feature pairs are close in the latent space. Cell-level contrastive learning ensures that mutual nearest neighbor (MNN) cell pairs are more closely aligned than other cell pairs, while feature-level contrastive learning ensures that feature pairs are more closely aligned compared to unrelated feature pairs.

For cell-level alignment, we treat the constructed MNN cell pairs in $M_{c}^{+}$ as positive pairs for contrastive learning, while all other cell pairs were regarded as negative pairs, with the set of negative pairs defined as $M_{c}^{-}$. The pairwise contrastive InfoNCE loss is then defined by the following equation:

$$L_{c}=-\frac{1}{\left|M_{c}^{+}\right|}\sum_{(i,j)\in M_{c}^{+}}\log\frac{\exp(\text{sim}(z_{i},z_{j})/\tau_{c})}{\sum_{(i,k)\in M_{c}^{-}}\exp(\text{sim}(z_{i},z_{k})/\tau_{c})},$$ (4)

where $\text{sim}(z_{i},z_{j})$ represents the cosine similarity between the embeddings *z*_*i*_ and *z*_*j*_, $\tau_{c}$ is a temperature constant, and |·| represents the number of elements in the set.

Similarly, for feature-level alignment, we treat the constructed feature pairs in $M_{f}^{+}$ as positive pairs and all other pairs as negative pairs, with the set of negative pairs defined as $M_{f}^{-}$. The contrastive loss for feature-level alignment is then defined as:

$$L_{f}=-\frac{1}{\left|M_{f}^{+}\right|}\sum_{(i,j)\in M_{f}^{+}}\log\frac{\exp(\text{sim}(y_{i},y_{j})/\tau_{f})}{\sum_{(i,k)\in M_{f}^{-}}\exp(\text{sim}(y_{i},y_{k})/\tau_{f})},$$ (5)

where $\tau_{f}$ is a temperature constant.

Overall, the total loss integrates three components: the reconstruction loss, the contrastive loss for MNN cell pairs across modalities, and the contrastive loss for feature pairs between the modalities,

$$L_{total}=L_{rec}+\alpha L_{c}+\beta L_{f},$$ (6)

where *α* and *β* are the trade-off parameters to balance the contributions of the different components of the total loss. Based on our experimental results, the parameters *α* and *β* are typically set to larger values, with default values of 1*e*4, while the temperature constants $\tau_{c}$ and $\tau_{f}$ are set to 0.5. By employing these steps, we ensure a robust and biologically informed integration of the scRNA-seq and gene activity score matrices or protein data, enhancing the alignment and interpretability of the integrated data.

#### Encoder and decoder architecture.

Our model employs dual encoders to learn compact latent representations for both cells and features across multiple modalities. Each encoder is a multi-layer fully connected network with two hidden layers (h_depth=2), projecting inputs into a latent space (latent_dim *d*). Each hidden layer consists of a linear transformation followed by batch normalization, LeakyReLU activation (negative slope 0.2), and dropout with a rate of 0.2. The input to the cell encoder is the PCA embedding of each cell, while the feature encoder takes the PCA embedding of each feature, the output corresponds to the latent embeddings of highly variable features.

The decoder reconstructs the original cell-by-feature matrix via a low-rank interaction between cell and feature embeddings:

$$\hat{X}=\text{softplus}(\text{scale})\cdot (ZY)+\text{bias},$$ (7)

where scale and bias are modality-specific learnable parameters. This design allows the model to capture the joint structure between cells and features effectively.

For training, mini-batch processing is used, with a default batch size of 256 for both cells and features to ensure memory-efficient computation. The model is optimized using the Adam optimizer with a learning rate of 0.0001 and a weight decay of 0.00005. The training runs for up to 1000 epochs with early stopping applied to prevent overfitting. This configuration allows the model to scale to large single-cell datasets while maintaining stable training dynamics.

### Visualization

To assess multi-omics integration methods, we employed several visualization techniques to evaluate performance. First, we used UMAP [64] to project the integrated data into a 2D space, coloring cells by modality or cell type. Although UMAP can intuitively reveal the uniformity of omics data distribution and the separation between cell types, it is important to note that UMAP should not be interpreted as a definitive measure of biological separations. We regard these visualizations as preliminary insights, with further analyses, such as clustering and quantitative assessments, providing more reliable evidence of biological distinctions.

To further evaluate biological consistency, we conducted trajectory analysis using PAGA [65] graphs. These graphs illustrate differentiation relationships and transition paths between cell types, with edge thickness representing the strength of inferred connections. Thicker edges suggest stronger potential transitions, and greater alignment with known differentiation trajectories indicates better preservation of biological structures.

### Evaluation metrics

Inspired by the previous related work [15,18] and a benchmarking study [66], we selected four quantitative metrics for integration evaluation, which are omics mixing, cell type conservation, label transfer accuracy and fraction of samples closer than the true match (FOSCTTM).

#### Omics mixing.

- **Graph Connectivity (GC)** evaluates the degree of omics mixing by measuring the largest connected component (LCC) within a k-nearest neighbor (kNN) graph for each cell type. A higher GC score indicates stronger connectivity across omics layers, reflecting better integration.
  $$\text{GC}=\frac{1}{T}\sum_{t=1}^{T}\frac{LCC_{t}}{n_{t}}$$
  where *LCC*_*t*_ represents the number of cells in the largest connected component for cell type *t*, *n*_*t*_ is the total number of cells of type *t*, and *T* denotes the total number of cell types. The GC score ranges from 0 to 1, with higher values indicating improved omics integration.
- **Seurat Alignment Score (SAS)** evaluates omics integration by measuring the proportion of k-nearest neighbors from the same omics layer for each cell.
  $$\text{A-score}=1-\frac{\overset{―}{x}-\frac{k}{V}}{k-\frac{k}{V}}$$
  where *k* is the number of neighbors, *V* is the number of omics, and $\overset{―}{x}$ is the average fraction of same-omics neighbors across all cells. SAS ranges from 0 to 1, with higher values indicating better alignment.
- **Average Silhouette Width across Omics (ASW-O)** quantifies omics mixing by computing the silhouette width for each cell based on omics layers. The score is defined as:
  $$\text{ASW-O}=\frac{1}{M}\sum_{j=1}^{M}\frac{1}{N_{j}}\sum_{i=1}^{N_{j}}1-|s_{\text{omic\_layer}}(i)|$$
  where $s_{\text{omic\_layer}}(i)$ is the silhouette width of cell *i* with respect to its omics layer. Specifically, it measures how well cell *i* is grouped with other cells from the same cell type and omics layer compared to cells from other omics layers within the same cell type. *N*_*j*_ is the number of cells in cell type *j*, and *M* is the total number of cell types. ASW-O ranges from 0 to 1, with higher values indicating better mixing of omics layers across cells.

Omics mixing quantifies the degree of integration across omics layers by aggregating multiple evaluation metrics. A higher omics mixing score indicates better integration and improved consistency between modalities. It is computed as:


$$\text{Omics Mixing}=\frac{\text{GC}+\text{SAS}+\text{ASW-O}}{3}.$$

#### Cell type conservation.

- **Mean Average Precision (MAP)** evaluates cell type resolution by measuring how well a cell’s k-nearest neighbors (kNN) match its true cell type. For each cell, the average precision (AP) is computed as the mean precision at each correctly matched neighbor. MAP is then obtained by averaging AP across all cells:
  $$\text{MAP}=\frac{1}{N}\sum_{i=1}^{N}\text{AP}(i)$$
  $$\text{AP}(i)=\frac{1}{m_{i}}\sum_{r=1}^{k}P(r)\cdot 𝕀(y_{i_{r}}=y_{i})$$
  $$P(r)=\frac{1}{r}\sum_{j=1}^{r}𝕀(y_{i_{j}}=y_{i})$$
  where *k* is the number of neighbors, *y*_*i*_ is the true cell type of cell *i*, $y_{i_{r}}$ is the cell type of its *r*-th nearest neighbor, and 𝕀(·) is an indicator function. *m*_*i*_ is the total number of correctly matched neighbors for cell *i*. MAP ranges from 0 to 1, with higher values indicating better cell type resolution.
- **Average Silhouette Width (ASW)** assesses cell type resolution by measuring how well each cell is clustered with others of the same type. The overall ASW is then obtained by averaging *s*(*i*) across all cells:
  $$\text{ASW}=\frac{1}{N}\sum_{i=1}^{N}s(i)$$
  where *s*(*i*) is the silhouette width for a cell *i*, ASW ranges from -1 to 1, with higher values indicating better separation between cell types and stronger clustering.
- **Normalized Mutual Information (NMI)** evaluates cell type resolution by quantifying the agreement between predicted and true cell type labels. It is defined as:
  $$\text{NMI}=\frac{2I(X,Y)}{H(X)+H(Y)}$$
  where *X* and *Y* represent the predicted and true cell type label distributions, *I*(*X*,*Y*) is the mutual information between them, and *H*(*X*) and *H*(*Y*) are their respective entropies. NMI ranges from 0 to 1, with higher values indicating better alignment between predicted and true labels.

The cell type conservation metric evaluates how well the integrated data preserve cell type identities. A higher score indicates better retention of biological distinctions. It is computed as:


$$\text{Cell Type Conservation}=\frac{\text{MAP}+\text{ASW}+\text{NMI}}{3}.$$

#### Label transfer accuracy.

- **Label Transfer Accuracy (LTA)** assesses integration quality in transfer learning by evaluating how well cell type labels transfer between omics. One omics dataset is used as the training set, while others serve as test sets. A classifier trained on the training set predicts cell types in the test set, and LTA is defined as the classification accuracy:
  $$\text{LTA}=\frac{\text{Correct Predictions}}{\text{Total Test Samples}}$$
  Higher LTA values indicate better cross-omics alignment. By default, a k-NN classifier with *k* = 5 is used, the largest dataset selected as the training set.

#### FOSCTTM.

- **Fraction of Samples Closer Than the True Match (FOSCTTM)** quantifies how well predicted cell correspondences align with the ground truth. For each cell in one omics, distances to all cells in the other omics are computed, and the fraction of cells closer than its true match is calculated as:
  $$\text{FOSCTTM}=\frac{1}{N}\sum_{i=1}^{N}\frac{\left|\{j|d(i,j)<d(i,i^{*})\}\right|}{M}$$
  where *d*(*i*,*j*) is the distance between cell *i* in one omics and cell *j* in the other, i^*^ is the true matched cell of *i*, and *M* is the total number of cells in the second omics. FOSCTTM ranges from 0 to 1, with lower values indicating better alignment.
  Note that FOSCTTM requires true one-to-one correspondences between cells across modalities and therefore cannot be applied to unpaired datasets.

### Datasets

We evaluated our method on six real multi-omics datasets: BMMC (paired) and PBMC (paired), which are paired multi-omics data with scRNA and scATAC modalities; Kidney, an unpaired dataset with snRNA and snATAC modalities; BMMC (unpaired) and PBMC (unpaired), which include unpaired scRNA and scATAC data; and BMCITE, a paired CITE-seq dataset that simultaneously measures scRNA and protein modalities. While some of the datasets are paired, we treated the paired data as unpaired, using the pairing information solely for quantitative evaluation purposes. A summary of these datasets, including the information of raw data and the dimensionality of the transformed matrices, is provided in Table 1.

**Table 1:** Summary of datasets used to evaluate the method. Each dataset includes pairing information and cell type annotations as ground truth for benchmarking.

Dataset	Modality	Sample Details	Source	#Cells	#Features	Ground Truth
**PBMC (paired)**	scRNA + scATAC	Peripheral blood mononuclear cells	10X Genomics [67]	9,631	29,095 genes, 107,194 peaks, 24,919 transformed features, 19,016 gene-transformed feature pairs	Paired data; cell type annotation
**PBMC (unpaired)**	scRNA + scATAC	Peripheral blood mononuclear cells	[32,68]	1,985(scRNA), 1,919(scATAC)	2,000 genes, 40,850 peaks, 1,931 transformed features, 1,735 gene-transformed feature pairs	Cell type annotation
**Kidney**	snRNA + snATAC	Human adult kidney cortex samples	GSE151302 [69]	19,985 (snRNA), 27,034 (snATAC)	33,538 genes, 196,113 peaks, 24,919 transformed features, 21,277 gene-transformed feature pairs	Cell type annotation
**BMMC (paired)**	scRNA + scATAC	Bone marrow mononuclear cells	GSE194122 [70,71]	6,224	13,431 genes, 116,490 peaks, 19,607 transformed features, 11,141 gene-transformed feature pairs	Paired data; cell type annotation
**BMMC (unpaired)**	scRNA + scATAC	Bone marrow mononuclear cells	GSE194122 [70,71]	6,224 (scRNA), 6,740 (scATAC)	13,431 genes, 116,490 peaks, 11,607 transformed features, 11,141 gene-transformed feature pairs	Unpaired data; cell type annotation
**BMCITE**	scRNA + protein	Bone marrow mononuclear cells	GSE194122 [70,71]	10,205 (s1d1/s1d2) or 16,451 (s1d2/s3d7)	13,953 genes, 134 proteins, 134 transformed features, 95 gene-transformed feature pairs	Paired data; cell type annotation

### Compared methods

We compared our method with 16 state-of-the-art methods, categorized into three groups: eight methods that integrate raw multi-omics data through nonlinear manifold alignment, six methods that use prior genomic information to transform features from other modalities into a gene activity score matrix, which shares a common feature space with scRNA-seq data, and two methods that leverage regulatory network information to construct a bridge between features across modalities. These methods are listed in Table 2, with detailed descriptions provided in Sect 3 of the S1 Text.

**Table 2:** The compared single cell multi-omics integration methods.

Input	Methods	Software
Raw scRNA-seq+scATAC-seq	MMD-MA [18]	Python
	UnionCom [15]	Python
	SCOT [72]	Python
	Pamona [16]	Python
	JointMDS [19]	Python
	scTopoGAN [17]	Python
	MultiVI [20]	Python
	scMoMaT [21]	Python
Raw scRNA-seq+gene activity score matrix	Seurat3 [24]	R
	LIGER [22]	R
	uniPort [68]	Python
	MultiMAP [23]	Python
	bindSC [25]	R
	scCross [26]	Python
Raw scRNA-seq+scATAC-seq + regulatory network	scDART [28]	Python
	GLUE [27]	Python

To ensure a fair comparison, we applied the same gene activity score transformation across all methods that required this preprocessing step. Specifically, we used either ArchR or Signac to generate gene activity score matrices from scATAC-seq data. The selected transformation method and parameter settings for each dataset of our BiCLUM method are summarized in S2 Table.

## Supporting information

S1 Fig UMAP visualizations of the integrated embeddings by different methods for PBMC (paired) data with cells colored based on omic types and cell types. (PDF)

S2 Fig PAGA trajectory visualizations for the PBMC (paired) data across different integration methods, where each node represents a cell type and the size is proportional to the number of cells in that type. Edges indicate potential lineage relationships, with the thickness representing the degree of connectivity between cell types. (PDF)

S3 Fig UMAP visualizations of the integrated embeddings by different methods for PBMC (unpaired) data with cells colored based on omic types and cell types. (PDF)

S4 Fig PAGA trajectory visualizations for the PBMC (unpaired) data across different integration methods, where each node represents a cell type and the size is proportional to the number of cells in that type. Edges indicate potential lineage relationships, with the thickness representing the degree of connectivity between cell types. (PDF)

S5 Fig UMAP visualizations of the integrated embeddings by different methods for Kidney data with cells colored based on omic types and cell types. (PDF)

S6 Fig PAGA trajectory visualizations for the Kidney data across different integration methods, where each node represents a cell type and the size is proportional to the number of cells in that type. Edges indicate potential lineage relationships, with the thickness representing the degree of connectivity between cell types. (PDF)

S7 Fig UMAP visualizations of the integrated embeddings by different methods for BMMC (paired) data with cells colored based on omic types and cell types. (PDF)

S8 Fig UMAP visualizations of the integrated embeddings by different methods for BMMC (unpaired) data with cells colored based on omic types and cell types. (PDF)

S9 Fig PAGA trajectory visualizations for the BMMC (paired) data across different integration methods, where each node represents a cell type and the size is proportional to the number of cells in that type. Edges indicate potential lineage relationships, with the thickness representing the degree of connectivity between cell types. (PDF)

S10 Fig UMAP visualizations of the integrated embeddings by different methods for BMCITE data for the two batches, s1d1 and s1d2, with cells colored based on omic types and cell types. (PDF)

S11 Fig UMAP visualizations of the integrated embeddings by different methods for BMCITE data for the two batches, s1d2 and s3d7, with cells colored based on omic types and cell types. (PDF)

S1 Table Summary of computational complexity and resource requirements for BiCLUM across datasets. (PDF)

S2 Table The parameter settings for different datasets. (PDF)

S1 Text Supplementary text. The file provides detailed descriptions of the generation of gene activity score matrices, clarifies the preprocessing procedures used in the BiCLUM method, and describes the compared methods.
(PDF)
