# Supplementary material for: BiCLUM: Bilateral contrastive learning for unpaired single-cell multi-omics integration
Source: PLoS Comput Biol. 2026 Feb 3;22(2):e1013932. doi: 10.1371/journal.pcbi.1013932 (PMC12904586; doi:10.1371/journal.pcbi.1013932)
Supplement: S4 Fig — (PDF) [file pcbi.1013932.s004.pdf]

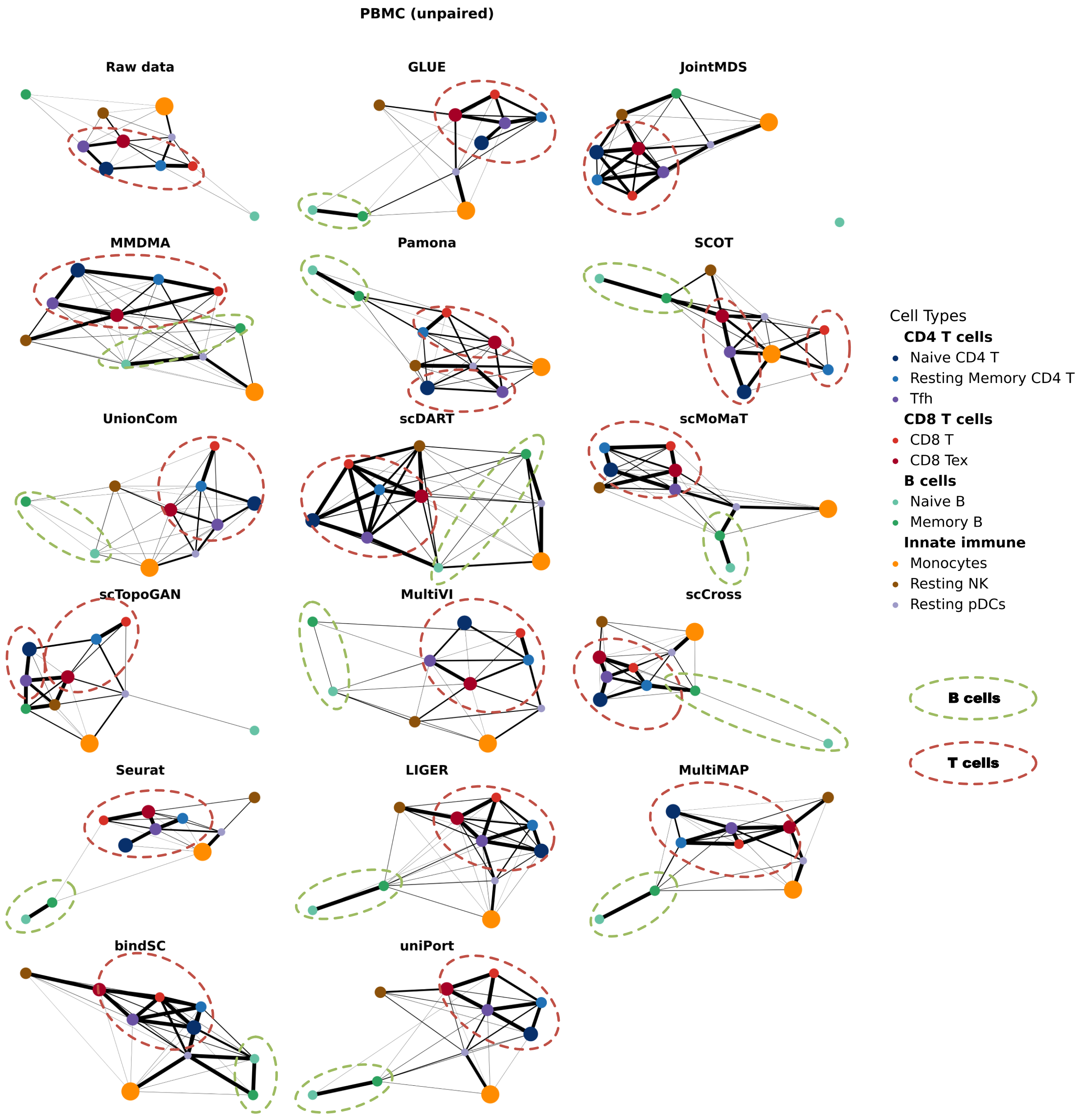

S4 Fig. PAGA trajectory visualizations for the PBMC (unpaired) data across different integration methods, where each node represents a cell type and the size is proportional to the number of cells in that type. Edges indicate potential lineage relationships, with the thickness representing the degree of connectivity between cell types.
