# Supplementary material for: BiCLUM: Bilateral contrastive learning for unpaired single-cell multi-omics integration
Source: PLoS Comput Biol. 2026 Feb 3;22(2):e1013932. doi: 10.1371/journal.pcbi.1013932 (PMC12904586; doi:10.1371/journal.pcbi.1013932)
Supplement: S8 Fig — (PDF) [file pcbi.1013932.s008.pdf]

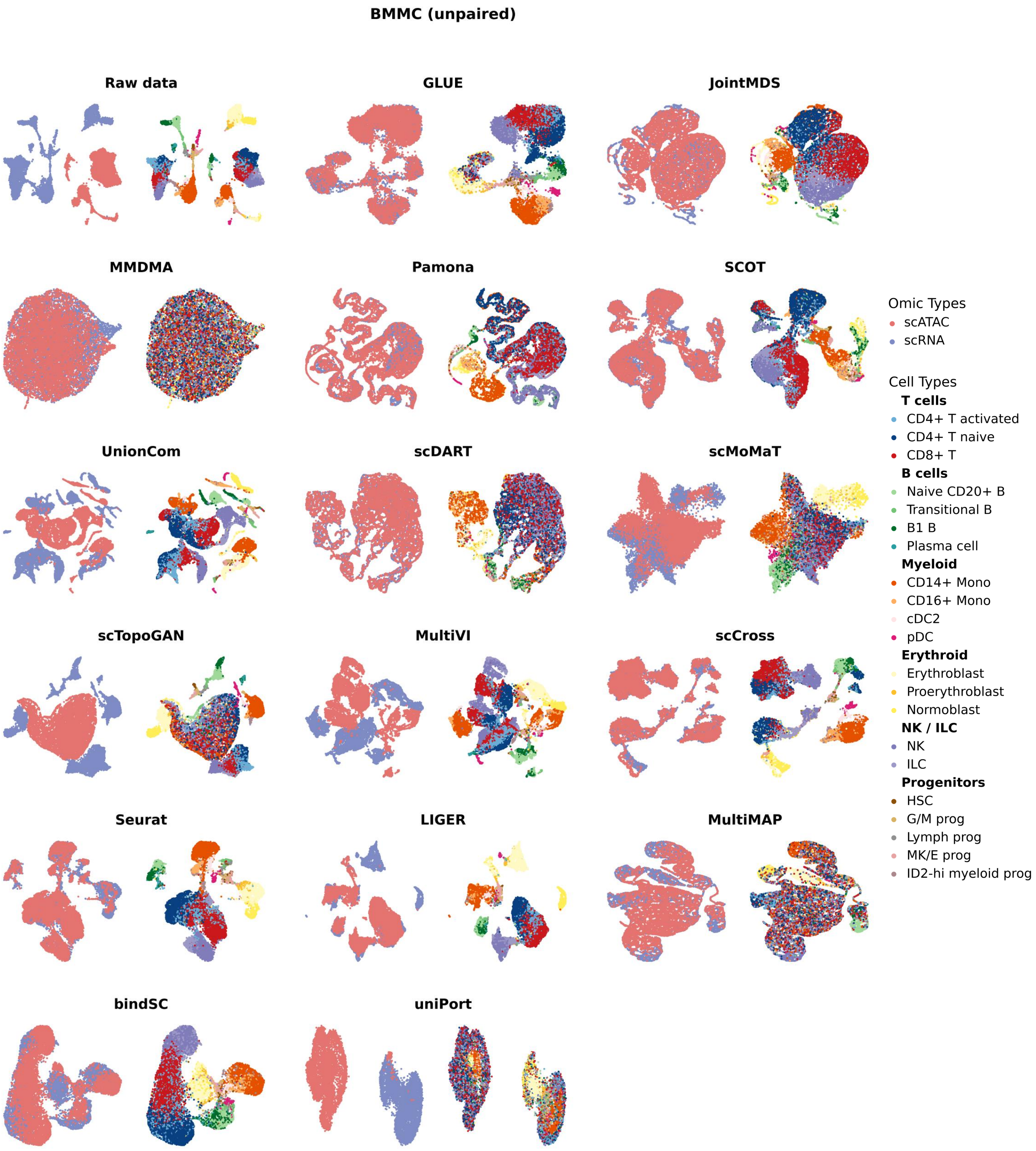

S8 Fig. UMAP visualizations of the integrated embeddings by different methods for BMMC (unpaired) data with cells colored based on omic types and cell types.
