# Supplementary material for: BiCLUM: Bilateral contrastive learning for unpaired single-cell multi-omics integration
Source: PLoS Comput Biol. 2026 Feb 3;22(2):e1013932. doi: 10.1371/journal.pcbi.1013932 (PMC12904586; doi:10.1371/journal.pcbi.1013932)
Supplement: S11 Fig — (PDF) [file pcbi.1013932.s011.pdf]

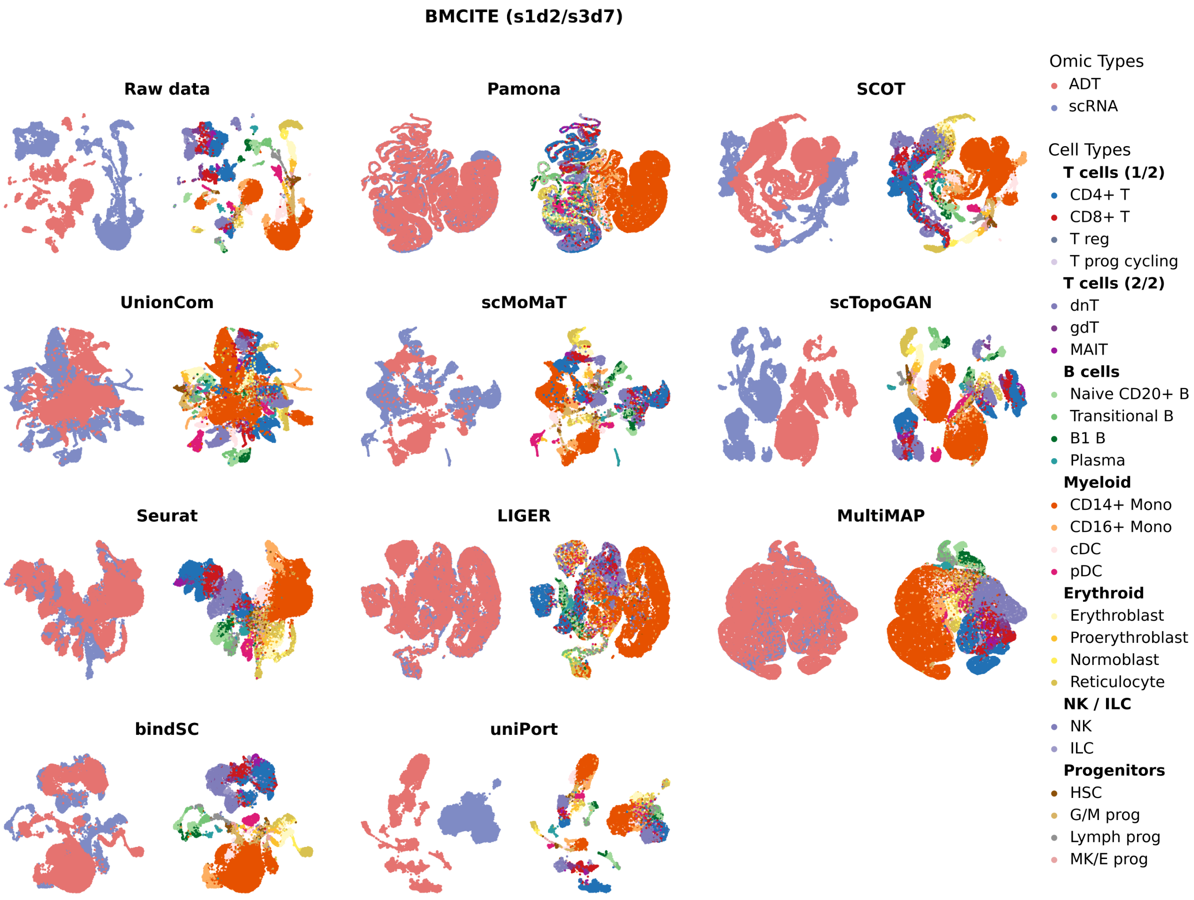

S11 Fig. UMAP visualizations of the integrated embeddings by different methods for BMCITE data for the two batches, s1d2 and s3d7, with cells colored based on omic types and cell types.
