# Supplementary material for: BiCLUM: Bilateral contrastive learning for unpaired single-cell multi-omics integration
Source: PLoS Comput Biol. 2026 Feb 3;22(2):e1013932. doi: 10.1371/journal.pcbi.1013932 (PMC12904586; doi:10.1371/journal.pcbi.1013932)
Supplement: S1 Table — (PDF) [file pcbi.1013932.s012.pdf]

S1 Table. Summary of computational complexity and resource requirements for BiCLUM across datasets.

Dataset	Runtime (s)	CPU usage (%)	RAM usage (%)	GPU load (%)	GPU memory (GB)	GPU power (W)
PBMC (unpaired)	402 ± 68	19.0 ± 0.9	18.3 ± 0.3	32 ± 9	1.08 ± 0.15	137 ± 30
PBMC (paired)	2245 ± 460	7.5 ± 0.1	15.2 ± 0.0	3 ± 4	1.63 ± 0.00	184 ± 2
Kidney	11409 ± 1969	16.2 ± 3.4	42.6 ± 7.1	27 ± 33	1.35 ± 1.00	120 ± 100
BMMC (paired)	1088 ± 138	7.7 ± 0.1	15.3 ± 0.2	38 ± 7	1.39 ± 0.00	183 ± 2
BMMC (unpaired)	1533 ± 312	21.1 ± 2.7	19.5 ± 3.0	50 ± 17	1.76 ± 0.53	199 ± 17
BMCITE(s1d1/s1d2)	732 ± 200	15.0 ± 4.0	20.7 ± 1.6	12 ± 18	0.84 ± 0.38	80 ± 72
BMCITE(s1d2/s3d7)	678 ± 204	5.7 ± 0.0	37.9 ± 0.0	0 ± 0	0.54 ± 0.00	30 ± 3
