# Supplementary material for: BiCLUM: Bilateral contrastive learning for unpaired single-cell multi-omics integration
Source: PLoS Comput Biol. 2026 Feb 3;22(2):e1013932. doi: 10.1371/journal.pcbi.1013932 (PMC12904586; doi:10.1371/journal.pcbi.1013932)
Supplement: S2 Table — (PDF) [file pcbi.1013932.s013.pdf]

S2 Table. The parameter settings for different datasets.

data	method/batches	α	β	τ_c	τ_f	k_{mnn}	d
PBMC (paired)	ArchR	1e4	1e4	0.5	10	200	50
PBMC (unpaired)	MAESTRO	1e4	1e4	0.5	0.5	100	50
kidney	ArchR	1e6	1e4	0.5	50	200	50
BMMC (paired)	Signac	1e4	1e4	0.5	0.5	200	50
BMMC (unpaired)	Signac	1e4	1e4	0.5	0.5	200	50
BMCITE	s1d1/s1d2	100	1e5	100	50	500	25
	s1d2/s3d7	100	1e5	100	50	500	25
