# Supplementary material for: BiCLUM: Bilateral contrastive learning for unpaired single-cell multi-omics integration
Source: PLoS Comput Biol. 2026 Feb 3;22(2):e1013932. doi: 10.1371/journal.pcbi.1013932 (PMC12904586; doi:10.1371/journal.pcbi.1013932)
Supplement: S1 Text — The file provides detailed descriptions of the generation of gene activity score matrices, clarifies the preprocessing procedures used in the BiCLUM method, and describes the compared methods. (PDF) [file pcbi.1013932.s014.pdf]

Supplementary Materials for "BiCLUM: Bilateral Contrastive Learning for Unpaired Single-Cell Multi-Omics Integration"

1 Methods for obtaining gene activity score matrices

Gene activity score can be used to infer the cell types for scATAC-seq datasets. There have been several methods analyse the scATAC-seq datasets by the gene activity score matrices.

- **ArchR** [1] creates a tile matrix based on a user-defined tile size and overlaps these tiles with a user-defined gene window. It calculates the distance from each tile to the gene body or TSS and identifies the tiles within the gene window that don't overlap with other genes. The distance is then converted to a weight using a user-defined accessibility model (default is e(-abs(distance)/5000) + e-1). To account for gene size differences, ArchR applies an inverse gene size weight (1/gene size), scaled linearly from 1 to a user-defined maximum (default: 5). The distance and gene size weights are multiplied by the number of Tn5 insertions in each tile and summed across all tiles within the gene window. This sum gives the gene score, which is normalized to a user-defined constant (default: 10,000).

- **Signac** [2] calculates gene activity scores by determining the number of fragments within genomic regions associated with each gene. The process starts by extracting gene coordinates and expanding them to include an upstream region of 2kb to cover regulatory elements in the promoter region. The FeatureMatrix() function is then used to count the fragments mapping to each gene region. This generates a gene activity matrix where rows represent cells, columns represent genes, and each cell value indicates the number of fragments in the corresponding gene region. Finally, the gene activity scores are log-normalized to standardize the data.

- **Cicero** [3] calculates gene activity scores by assessing the chromatin accessibility at the promoter region and the regulatory potential of nearby chromatin peaks. It uses a correlation-based model to quantify the relationship between distal regulatory regions and the target gene's promoter. Specifically, Cicero identifies chromatin interactions by measuring the co-accessibility between the promoter and distal peaks, where high co-accessibility indicates potential gene activation.

- **Gene Scoring** [4] assigns each gene an accessibility score by aggregating the chromatin accessibility peaks around its transcription start site (TSS). The method applies an exponential decay function to weight peaks according to their distance from

the TSS, with closer peaks given higher weights. This scoring system reflects the regulatory influence of proximal enhancers or other elements near the TSS on gene activity.

- **MAESTRO** [5] computes a gene activity score as a weighted sum of nearby cis-regulatory elements (REs), where the weight is determined by an exponentially decaying function of the distance between the regulatory elements (such as enhancers) and the target gene. This method quantifies the influence of nearby regulatory elements on gene activity, with closer elements having a stronger regulatory effect.

- **cisTopic** [6] applies Latent Dirichlet Allocation (LDA), a Bayesian topic modeling technique commonly applied in natural language processing, to identify cell states based on the distribution of topics across cells, and to explore cis-regulatory regions through the distribution of regions across topics. By combining the topic-cell distribution with the region-topic distribution, cisTopic calculates the probability of each region being active in a given cell (i.e., the predictive distribution). Finally, the gene activity score for each gene is derived by summing the probabilities of regions associated with that gene, typically those linked to known marker genes.

- **SnapATAC2** [7] Instead of fragment counts, SnapATAC2 uses counts of Tn5 insertion sites within gene bodies and normalizes these counts using a log-transformed count-per-million reads (CPM) approach. Additionally, SnapATAC2 employs a method based on Markov Affinity Graphs for data imputation and smoothing. This method takes into account the proximity of Tn5 insertion sites, weights the distance of these sites from the transcription start site (TSS), and also considers the influence of distal regulatory elements.

2 Data preprocessing

To integrate scRNA-seq data with the gene activity score matrix from scATAC-seq data, both sharing the same gene features, we utilize a structured preprocessing pipeline implemented with the Scanpy package. The process starts by identifying HVGs using the *scanpy.pp.highly_variable_genes* function for each matrix individually. These HVGs from both datasets are then combined to form a unified set of HVGs for the integration. The subsequent normalization steps are performed separately for each dataset. Specifically, we use the *scanpy.pp.normalize_total* function for normalization, followed by log transformation with *scanpy.pp.log1p*, and scaling the data using *scanpy.pp.scale*. Finally, to reduce

dimensionality, PCA embeddings are computed using the *scanpy.tl.pca* function, setting the number of principal components (npca) to 100 by default. This workflow ensures that both datasets are processed consistently, allowing for a more effective integration of the scRNA-seq and gene activity score data in subsequent analyses.

For integrating scRNA and single-cell protein modalities, the preprocessing steps for each modality are designed to handle their specific characteristics. For the protein data, we follow the standard procedures used in Seurat. The data is normalized using the NormalizeData() function, followed by scaling with the ScaleData() function. After these steps, PCA is applied to reduce dimensionality using the RunPCA() function. For the scRNA data, preprocessing can be performed using either the Scanpy or Seurat package. If using Seurat, the functions NormalizeData(), FindVariableFeatures(), ScaleData(), and RunPCA() are applied sequentially. Alternatively, using Scanpy, the preprocessing procedure is similar to that of scRNA and gene activity score matrix of scATAC data. Note that scRNA data is typically high-dimensional and sparse, which necessitates the selection of highly variable genes to reduce noise and improve the integration process. In contrast, protein data usually has fewer features (hundreds at most), which generally does not require feature selection, as its dimensionality is much lower.

3 Compared methods

The existing methods can be roughly divided into three types:

- **Learn latent embeddings for original omics layers via nonlinear manifold alignment: MMD-MA [8], UnionCom [9], Scot [10], Pamona [11], JointMDS [12], scTopoGAN [13], MultiVI [14], scMoMaT [15].**
  - MMD-MA integrates cells measured in different ways into a common latent space based on the Maximum Mean Discrepancy (MMD), preserving the structure of the original high-dimensional dataset in the low-dimensional embedding.
  - UnionCom is a two-step method which first finds the correspondence between the cells from different omics by aligning the corresponding kernels, and then find low-dimensional representations by preserving the correspondence and local structure.
  - JointMDS integrates the multidimensional scaling (MDS) and Wasserstein Procrustes analysis into a joint optimization problem to simultaneously learn the

low-dimensional latent embedding of each omic and the correspondence between cells from two different omics.

  - Pamona develops a partial Gromov-Wasserstein optimal transport framework to divide cells in different omics into shared and dataset-specific cells, which can align the cells in a common low-dimensional space while preserving shared and dataset-specific structures.
  - SCOT is also a Gromov-Wasserstein-based optimal transport method to align single-cell multi-omics datasets. The method first constructs a k-NN graph for each omic and finds a probabilistic coupling between the cells of each omic to minimize the distance between the graph distance matrices generated by the k-NN graph. At last, the coupling matrix is used to perform alignment by projecting one single cell dataset onto another single cell dataset.
  - scTopoGAN uses topological autoencoders to obtain latent representations of each modality separately. A topology-guided Generative Adversarial Network then aligns these latent representations into a common space.
  - MultiVI is a deep generative model that learns a shared latent representation across multiple omics layers through nonlinear manifold alignment. Each modality is modeled with a modality-specific encoder–decoder pair under a unified variational autoencoder framework, and an additional alignment loss encourages the latent embeddings of paired cells from different modalities to be close in the latent space.
  - scMoMaT is a matrix tri-factorization–based framework designed for single-cell mosaic integration and multi-modal biomarker detection. It jointly learns shared low-dimensional representations of cells and features across partially overlapping batches and modalities by decomposing each observed matrix into cell-specific, feature-specific, and modality–batch-specific latent factors. Through this unified factorization, scMoMaT aligns heterogeneous omics datasets while simultaneously identifying modality-specific biomarkers (e.g., genes, chromatin regions, or proteins) that define each cell population.

- **Convert multimodality data into one common feature space based on prior knowledge : Seurat3 [16], LIGER [17], uniPort [18], MultiMAP [19], bindSC [20], scCross [21].**

  - Seurat3 is an alignment workflow integrating single-cell multi-omics datasets with CCA amd MNN procedures.

  - LIGER identifies shared and dataset-specific factors through integrative non-negative matrix factorization.
  - uniPort is also a method that also makes use of the optimal transport strategy and the omic-specific features are considered in the method.
  - MultiMAP learns a low-dimensional representation of multimodal data based on the UMAP framework.
  - bindSC employs a gene activity matrix as a bridge to link multiple omics datasets and performs canonical correlation analysis (CCA) to achieve alignment at both the feature and cell levels.
  - scCross is another deep generative framework that combines modality-specific VAEs, GAN-based regularization, and mutual nearest neighbor (MNN) anchors to achieve cross-modal alignment and enable cross-modal data generation and in silico perturbations. scCross also integrates gene-set score vectors (gene activity score matrix) as biological priors to enhance alignment quality.

- **Make use of the information of regulatory network during the integration process: scDART [22], GLUE [23].**
  - GLUE learns latent embeddings by constructing a knowledge-guided graph to link features across modalities and employs an adversarial network to align cells.
  - scDART also uses prior knowledge to define a linkage matrix between features across modalities and aligns the datasets through MMD.

References

[1] Jeffrey M Granja, M Ryan Corces, Sarah E Pierce, S Tansu Bagdatli, Hani Choudhry, Howard Y Chang, and William J Greenleaf. Archr is a scalable software package for integrative single-cell chromatin accessibility analysis. *Nature genetics*, 53(3):403–411, 2021.

[2] Tim Stuart, Avi Srivastava, Shaista Madad, Caleb A Lareau, and Rahul Satija. Single-cell chromatin state analysis with signac. *Nature methods*, 18(11):1333–1341, 2021.

[3] Hannah A Pliner, Jonathan S Packer, José L McFaline-Figueroa, Darren A Cusanovich, Riza M Daza, Delasa Aghamirzaie, Sanjay Srivatsan, Xiaojie Qiu, Dana Jackson, Anna Minkina, et al. Cicero predicts cis-regulatory dna interactions from single-cell chromatin accessibility data. *Molecular cell*, 71(5):858–871, 2018.

[4] Caleb A Lareau, Fabiana M Duarte, Jennifer G Chew, Vinay K Kartha, Zach D Burkett, Andrew S Kohlway, Dmitry Pokholok, Martin J Aryee, Frank J Steemers, Ronald Lebofsky, et al. Droplet-based combinatorial indexing for massive-scale single-cell chromatin accessibility. *Nature Biotechnology*, 37(8):916–924, 2019.

[5] Chenfei Wang, Dongqing Sun, Xin Huang, Changxin Wan, Ziyi Li, Ya Han, Qian Qin, Jingyu Fan, Xintao Qiu, Yingtian Xie, et al. Integrative analyses of single-cell transcriptome and regulome using maestro. *Genome biology*, 21(1):1–28, 2020.

[6] Carmen Bravo González-Blas, Liesbeth Minnoye, Dafni Papasokrati, Sara Aibar, Gert Hulselmans, Valerie Christiaens, Kristofer Davie, Jasper Wouters, and Stein Aerts. cistopic: cis-regulatory topic modeling on single-cell atac-seq data. *Nature methods*, 16(5):397–400, 2019.

[7] Bing Ren, Kai Zhang, Nathan Zemke, and Ethan Armand. Snapatac2: a fast, scalable and versatile tool for single-cell omics analysis. *bioRxiv*, pages 2023–09, 2023.

[8] Ritambhara Singh, Pinar Demetci, Giancarlo Bonora, Vijay Ramani, Choli Lee, He Fang, Zhijun Duan, Xinxian Deng, Jay Shendure, Christine Disteche, et al. Unsupervised manifold alignment for single-cell multi-omics data. In *Proceedings of the 11th ACM International Conference on Bioinformatics, Computational Biology and Health Informatics*, pages 1–10, 2020.

[9] Kai Cao, Xiangqi Bai, Yiguang Hong, and Lin Wan. Unsupervised topological alignment for single-cell multi-omics integration. *Bioinformatics*, 36(Supplement_1):i48–i56, 2020.

[10] Pinar Demetci, Rebecca Santorella, Björn Sandstede, William Stafford Noble, and Ritambhara Singh. Scot: Single-cell multi-omics alignment with optimal transport. *Journal of Computational Biology*, 29(1):3–18, 2022.

[11] Kai Cao, Yiguang Hong, and Lin Wan. Manifold alignment for heterogeneous single-cell multi-omics data integration using pamona. *Bioinformatics*, 38(1):211–219, 2022.

[12] Dexiong Chen, Bowen Fan, Carlos Oliver, and Karsten Borgwardt. Unsupervised manifold alignment with joint multidimensional scaling. *arXiv preprint arXiv:2207.02968*, 2022.

[13] Akash Singh, Marcel JT Reinders, Ahmed Mahfouz, and Tamim Abdelaal. sctopogan: unsupervised manifold alignment of single-cell data. *bioRxiv*, pages 2022–04, 2022.

[14] Tal Ashuach, Mariano I Gabitto, Rohan V Koodli, Giuseppe-Antonio Saldi, Michael I Jordan, and Nir Yosef. Multivi: deep generative model for the integration of multimodal data. *Nature Methods*, 20(8):1222–1231, 2023.

[15] Ziqi Zhang, Haoran Sun, Ragunathan Mariappan, Xi Chen, Xinyu Chen, Mika S Jain, Mirjana Efremova, Sarah A Teichmann, Vaibhav Rajan, and Xiuwei Zhang. scmomat jointly performs single cell mosaic integration and multi-modal bio-marker detection. *Nature Communications*, 14(1):384, 2023.

[16] Tim Stuart, Andrew Butler, Paul Hoffman, Christoph Hafemeister, Efthymia Papalexi, William M Mauck, Yuhan Hao, Marlon Stoeckius, Peter Smibert, and Rahul Satija. Comprehensive integration of single-cell data. *Cell*, 177(7):1888–1902, 2019.

[17] Jialin Liu, Chao Gao, Joshua Sodicoff, Velina Kozareva, Evan Z Macosko, and Joshua D Welch. Jointly defining cell types from multiple single-cell datasets using liger. *Nature protocols*, 15(11):3632–3662, 2020.

[18] Kai Cao, Qiyu Gong, Yiguang Hong, and Lin Wan. A unified computational framework for single-cell data integration with optimal transport. *Nature Communications*, 13(1):7419, 2022.

[19] Mika Sarkin Jain, Krzysztof Polanski, Cecilia Dominguez Conde, Xi Chen, Jongeun Park, Lira Mamanova, Andrew Knights, Rachel A Botting, Emily Stephenson, Muzlifah Haniffa, et al. Multimap: dimensionality reduction and integration of multimodal data. *Genome biology*, 22(1):1–26, 2021.

[20] Jinzhuang Dou, Shaoheng Liang, Vakul Mohanty, Qi Miao, Yuefan Huang, Qingnan Liang, Xuesen Cheng, Sangbae Kim, Jongsu Choi, Yumei Li, et al. Bi-order multimodal integration of single-cell data. *Genome biology*, 23(1):1–25, 2022.

[21] Xiuhui Yang, Korenk. Mann, Hao Wu, and Jun Ding. sccross: a deep generative model for unifying single-cell multi-omics with seamless integration, cross-modal generation, and in silico exploration. *Genome Biology*, 25(1):1–34, 2024.

[22] Ziqi Zhang, Chengkai Yang, and Xiuwei Zhang. scdart: integrating unmatched scrna-seq and scatac-seq data and learning cross-modality relationship simultaneously. *Genome Biology*, 23(1):139, 2022.

[23] Zhi-Jie Cao and Ge Gao. Multi-omics single-cell data integration and regulatory inference with graph-linked embedding. *Nature Biotechnology*, 40(10):1458–1466, 2022.
